# Human ostension enhances attentiveness but not performance in domestic pigs

**DOI:** 10.1038/s41598-025-00511-7

**Published:** 2025-05-09

**Authors:** Kimberly Brosche, Ariane Veit, Paula Pérez Fraga, Marianne Wondrak, Attila Andics, Zsófia Virányi

**Affiliations:** 1 Messerli Research Institute, University of Veterinary Medicine Vienna, Medical University of Vienna, University of Vienna, Veterinärplatz 1, Vienna, 1210 Austria; 2 Neuroethology of Communication Lab, Department of Ethology, Eötvös Loránd University, Budapest, Hungary; 3 Gut Aiderbichl, Berg 20, Henndorf am Wallersee, 5302 Austria; 4 ELTE NAP Canine Brain Research Group, Budapest, Hungary

**Keywords:** Ostension, Domestic pig, *Sus scrofa domesticus*, Human-animal communication, Domestication, Psychology, Animal behaviour

## Abstract

Humans convey their communicative intentions ostensively, e.g., calling others’ name and establishing eye-contact. Also when interacting with animals, humans use ostension. In some companion-animal species, ostension increases attentiveness and/or alters how animals process human-provided information. However, it is unclear whether domesticated species selected for purposes other than companionship, like pigs, would also be sensitive. We investigated whether pigs are sensitive to human ostension and whether experience with humans modulates pigs’ sensitivity. Fifty-four pigs with varying experience with humans completed ostensive and non-ostensive versions of (1) an object-choice task with directional cues, (2) an A-not-B-task, and (3) a detour task with human demonstrations. We expected pigs to respond to human ostension with increased attentiveness and possibly altered performance. Further, if sensitivity to ostension increases with experience with humans, more intensively socialized pigs should be more attentive and more likely to change in performance than less socialized ones. Results suggest that pigs were more attentive to ostensive than non-ostensive detour demonstrations. Otherwise ostension did not affect attentiveness or performance. This suggests that pigs might be less inclined than species selected for companionship to process human-provided information differently. Attentiveness, however, seems to be enhanced by ostension also in animals selected for production purposes. Moreover, we found that living conditions and experience, e.g., training, influenced pigs’ attentiveness and performance, independently of ostension. These findings highlight the influence of training experience and enrichment on pigs’ cognitive performance.

**Supplementary Information:**

The online version contains supplementary material available at 10.1038/s41598-025-00511-7.

## Introduction

One of the unique characteristics of human communication is ostension. Communicating ostensively involves not only drawing attention to the information but also transmitting one’s communicative intention to inform others^1,2^. To convey this intention to communicate, senders might perform exaggerated movements or explicitly address others by name, or they may establish eye-contact before performing an action aimed at informing others^3–5^. Sensitivity to ostension develops early in humans, as it can substantially change even infants’ perception of the context: they interpret information provided in an ostensive manner as referential and/or generalizable beyond the specific context^3,6,7^. As such, in human communication, ostension plays a crucial role in facilitating teaching^6,8^ and cooperation^4^. Importantly though, humans do not only engage in ostensive communication with human infants or adults but frequently also with non-human animals. For instance, adult humans have been shown to use a similar speech register when addressing their pets than when addressing human infants^9,10^. Even in the absence of active production of ostensive signals, sensitivity to ostension is presumably adaptive for both human infants and non-human domesticated animals living in close proximity with humans as it facilitates learning from human adults^4^.

However, it remains unclear whether sensitivity to ostension is sufficiently adaptive for all domesticated animals or solely for those specifically selected for companionship. The reason for this is that, so far, domesticated species’ sensitivity has exclusively been assessed in companion animal species such as dogs, horses, and cats. This is insufficient for two reasons. First, dogs, horses and cats have ample experience with humans. Second, they are not only domesticated but, at least dogs and horses, are also specifically selected for companionship and cooperation with humans. Therefore, relying exclusively on these species, it is impossible to determine the extent to which domestication, selection for companionship^11,12^ and intensive socialization with humans contribute to animals’ selective sensitivity to ostension. To address this question, data from domesticated species selected for other purposes, such as meat production, as well as from different individuals from the same species that were socialized with humans to a varying degree, are called for (see also^13^).

Here we suggest that domestic pigs *(Sus scrofa domesticus)* are highly suitable subjects to address this question. On the one hand, pigs are domesticated but were not selected for companionship^14^. On the other hand, pigs are nowadays frequently used in research and have also gained popularity as companion and zoo animals in recent years^15^. This variety of different living environments and, concomitantly, degrees of socialization with humans, makes pigs ideal candidates to investigate the impact of exposure to human communication on domesticated animals’ sensitivity to human ostension.

Previous studies have compared animals’ reactions to ostensive and non-ostensive signals in three established paradigms: (1) pointing or gaze following tasks^2,16,17^, (2) an A-not-B task^18–20^, and (3) a detour task^21^. Using these three paradigms, we aimed to investigate whether pigs show a selective sensitivity to human ostensive communication in (a) their attention compared to non-ostensive contexts and (b) in their performance in these tasks. While pigs could be affected merely on the level of attentiveness, without ostension modulating the way they use the information presented (i.e., no effect on their performance in the tasks see^22^, ostension could also help pigs perceive the human-provided information differently^19,21,23^, altering their performance in the task, beyond an attention-enhancing effect. Therefore, we assessed whether human ostensive signals (compared with similar, non-ostensive ones) lead to (a) increased attentiveness^22^ and/or (b) altered performance when attentiveness is controlled for^7,18,19,21,23,24^, by employing the three tasks described below.

First, the effect of ostension on animals’ ability to follow human directional pointing or gaze cues has been studied in object-choice tasks. In such tasks, the experimenter points or gazes at the baited one of two locations (e.g., bowls) – either ostensively or non-ostensively. In the ostensive condition, the experimenter might call the animal’s name before performing the pointing or gazing cue. In the non-ostensive condition, the experimenter instead seemingly accidentally points or gazes at the location, e.g., while pretending to massage their shoulder or check a wrist watch^2,16^. Applying such paradigms, it has been shown that dogs^16^ (*Canis lupus familiaris*) and horses^17^ (*Equus caballus*) are more receptive to ostensive directional cues than non-ostensive ones. In contrast, cats (*Felis catus*) show a difference in attentiveness (looking at the experimenter), but not in success at following the directional cues, between ostensive and non-ostensive versions of an object-choice task^22^. In the present study, pigs were exposed to the experimenter’s directional gaze and body-orientation cues, which were preceded by either ostensive or non-ostensive attention-getting. If pigs are sensitive to ostension, we would expect them to be more attentive to the ostensively-presented cues and/or to be more successful in following ostensive than non-ostensive directional cues to the baited one of two bowls^16^, even when attentiveness is controlled for.

Second, sensitivity to ostension has been investigated in the A-not-B task. In A-not-B tasks, one of two locations (“A”) is baited conspicuously over several trials, before the target’s location is switched to the other side (“B”). Even though they should “know better” after having observed the baiting process, both human infants^7^ and dogs^18^ continue to search in the initial, now incorrect location (“A”), committing the so-called “A-not-B” error. Importantly, both species perseverate significantly more after ostensive hiding (e.g., when the experimenter calls their name before hiding the target) than after non-ostensive hiding, indicating that other mechanisms such as generalization or compliance may be at play^16,19^. In contrast to infants and dogs, cats made fewer mistakes in the ostensive than non-ostensive trials, likely due to ostension merely increasing subjects’ attentiveness, enabling them to better follow the hiding to the new location^20^. Analogously, if pigs are sensitive to human ostension, we would expect them to pay more attention to the ostensive hiding in the A-not-B task. In addition, despite paying close attention, they should commit the A-not-B error more, i.e., have a lower success rate in the B trials, in the ostensive condition than in the non-ostensive condition.

Finally, animals’ behavior in detour tasks with human demonstrations can be used to assess sensitivity to ostension. In these tasks, a target (e.g., a food reward) is placed behind a fence and animals must detour this fence to reach the target. To probe sensitivity to ostension, a human experimenter can demonstrate the route around the fence – ostensively in one condition and non-ostensively in another condition. In the ostensive condition, the experimenter might call the animal’s name, while, in the non-ostensive condition they might talk to themselves or recite a text non-ostensively. In dogs, only ostensive human demonstrations, but not non-ostensive ones, significantly reduced dogs’ latency to detour the fence and reach the target relative to unaided trials without a demonstration^21^. Attentiveness, measured as the visual orientation of the dog, did not differ between conditions and can hence not account for this effect of ostension. In the present study, if pigs are sensitive to human ostension, we would expect their attentiveness to the detour demonstration to be higher and/or their attentiveness-independent latency to reach the target behind the fence to be shorter after ostensive than after non-ostensive demonstrations.

In addition to the question whether also pigs, being a domesticated species not selected for companionship, are sensitive to human ostensive communication, we aimed to explore whether pigs’ potential sensitivity to ostension could be modulated by different degrees of experience with humans. To explore this possibility, we subjected three different pig populations (varying in their degree of exposure to human communication) to the three tasks. The three groups of pigs were (a) miniature pigs kept as companion animals in human households (“companion pigs”), (b) free-ranging pigs kept in a kin-based sounder for behavioral research (“lab pigs”), and (c) breeding sows housed at a commercial pig farm (“commercial pigs”). We expected that, if experience with humans has an impact on sensitivity to ostension, we would find differences in sensitivity across the three pig groups with differential exposure to human communication. Precisely, we expected the commercial pigs to be least and the companion pigs to be most sensitive to ostensive communication, with lab pigs taking an intermediate position.

## Results

We tested eight companion pigs (4 f, 4 m), 31 lab pigs (16 f, 15 m), and 15 commercial pigs (15 f) in ostensive and non-ostensive versions of (1) an object-choice task with directional gaze and body orientation cues, (2) an A-not-B task, and (3) a detour task with human demonstrations, using a within-subject design. For each task, we measured the proportion of time pigs were attentive to the experimenter during the demonstrations, as well as their performance in the task. Attentiveness was defined as the pig’s snout being oriented towards the experimenter (for details see “Methods” section), without the pig being visibly occupied with something else or looking up at the owner/assistant next to the start box. In all cases, we analyzed whether condition (ostensive or non-ostensive), pig group (companion, lab or commercial pig) or the interaction between the two had a significant effect on pigs’ attentiveness using full-null model comparisons. In all cases, the term “null-model” is used to refer to a model lacking the predictors of interest, and their interactions but containing the control predictors and random effects structure. To be able to draw conclusions about pigs’ use of the demonstrations, we analyzed pigs’ success in the tasks (correct choices in the object-choice and the A-not-B task, and latency in the detour task) by comparing full models, containing the fixed effects condition, pig group and trial (only for the A-not-B task and the detour task) as well as any possible interactions between those, with null models lacking these effects, while controlling for pigs’ attentiveness. Whenever a full-null model comparison revealed a significant difference, we applied step-wise model reduction to identify significant interactions or main effects as well as pairwise comparisons of the levels of significant predictors where applicable.

### Attentiveness—object-choice task

**Fig. 1:**
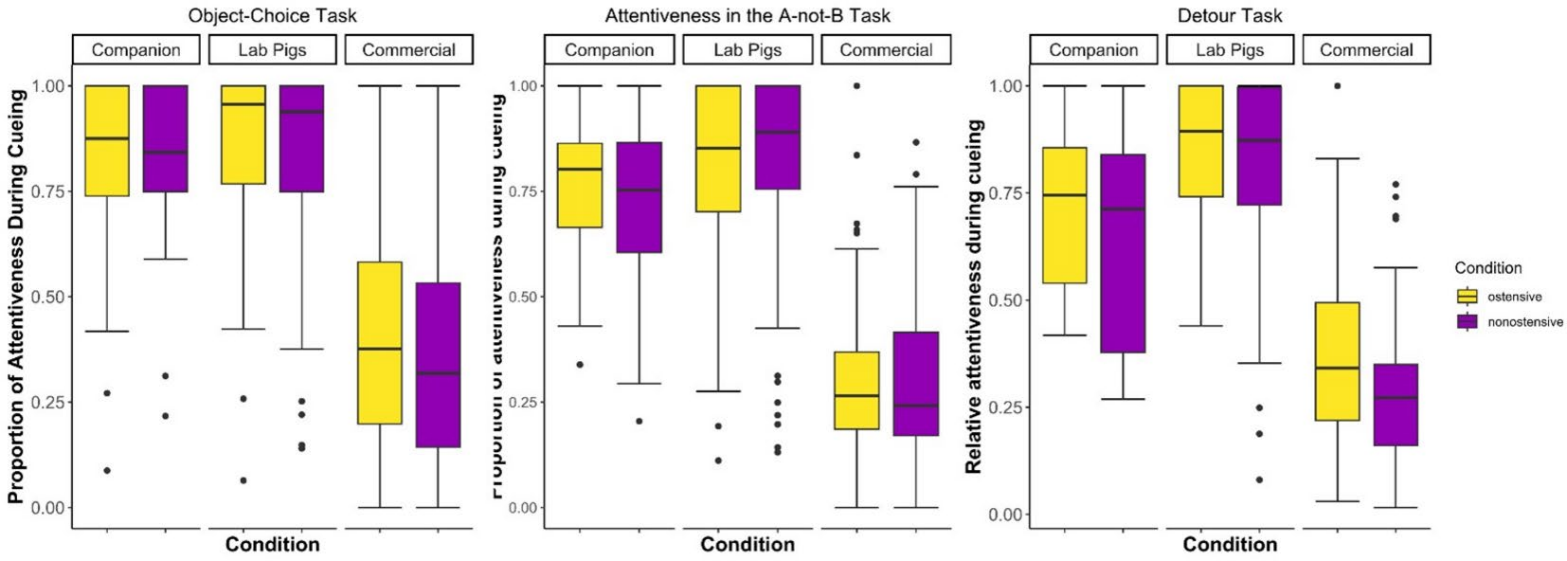
Proportion of time the pigs were attentive during cueing in the three tasks (**a** Object-Choice Task, **b**: A-not-B Task, **c**: Detour Task) for the three pig groups in the two conditions (ostensive in yellow, non-ostensive in purple).

When analyzing the pigs’ attentiveness in the object-choice task, the full model provided a significantly better fit to the data than the null model (*n* = 363 trials across 54 pigs, *χ²* = 56.84, *df* = 5, *p* < 0.001, see Table S6 for full model results). The comparison of reduced models revealed that the two-way interaction of condition and group itself was not significant (*χ²* = 0.22, *df* = 2, *p* = 0.89). However, there was a tendency for pigs to be more attentive in the ostensive condition (*χ²* = 605.22, *df* = 1, *p* = 0.085) and the main effect of group was significant (*χ²* = 54.31, df = 2, *p* < 0.001), see Fig. 1. Pairwise comparisons of the pig groups revealed that commercial pigs were significantly less attentive than companion (*z* = -6.62, *p* < 0.001) and lab pigs (*z* = -9.42, *p* < 0.001), with no significant difference between companion and lab pigs (*z* = -0.74, *p* = 0.74), see Table S7.

### Attentiveness—A-not-B task

The full model for the pigs’ attentiveness in the A-not-B test provided a significantly better fit to the data than the null model (*n* = 498 trials across 50 pigs, *χ²* = 126.57, *df* = 5, *p* < 0.001, for full model results see Table S8). While neither the two-way interaction (*χ²* = 0.552, *df* = 2, *p* = 0.76), nor condition (*χ²* = 0.572, *df* = 1, *p* = 0.45) was significant, group had a significant effect on the pigs’ attentiveness (*χ²* = 56.519, *df* = 2, *p* < 0.001, see Fig. 1). Pairwise comparisons revealed that commercial pigs’ attentiveness was significantly lower than that of companion (*z* = -6.111, *p* < 0.001) and lab pigs (*z* = -10.067, *p* < 0.001). Lab pigs also tended to be more attentive than companion pigs (*z* = -2.226, *p* = 0.067), see Table S9.

### Attentiveness—detour task

In the analysis targeting pigs’ attentiveness in the demonstration trials of the detour task, the full model provided a significantly better fit to the data than the null model (*n* = 298 trials across 50 pigs, *χ²* = 67.00, *df* = 5, *p* < 0.001, see Fig. 1, for full model results see Table S10). The two-way interaction was not significant (*χ²* = 0.650, *df* = 2, *p* = 0.72), but pigs were significantly more attentive in the ostensive than in the non-ostensive condition (*χ²* = 5.165, *df* = 1, *p* = 0.02) and the pig groups differed significantly (*χ²* = 61.315, *df* = 2, *p* < 0.001). When conducting pairwise comparisons between the pig groups, we detected significant differences for all pairs (commercial and companion pigs: *z* = -5.720, *p* < 0.001; commercial and lab pigs: *z* = -10.385, *p* < 0.001; companion and lab pigs: *z* = -3.084, *p* = 0.006, see Table S11). That is, lab pigs were most attentive, followed by companion and, lastly, commercial pigs.

### Choice—object-choice task

**Fig. 2:**
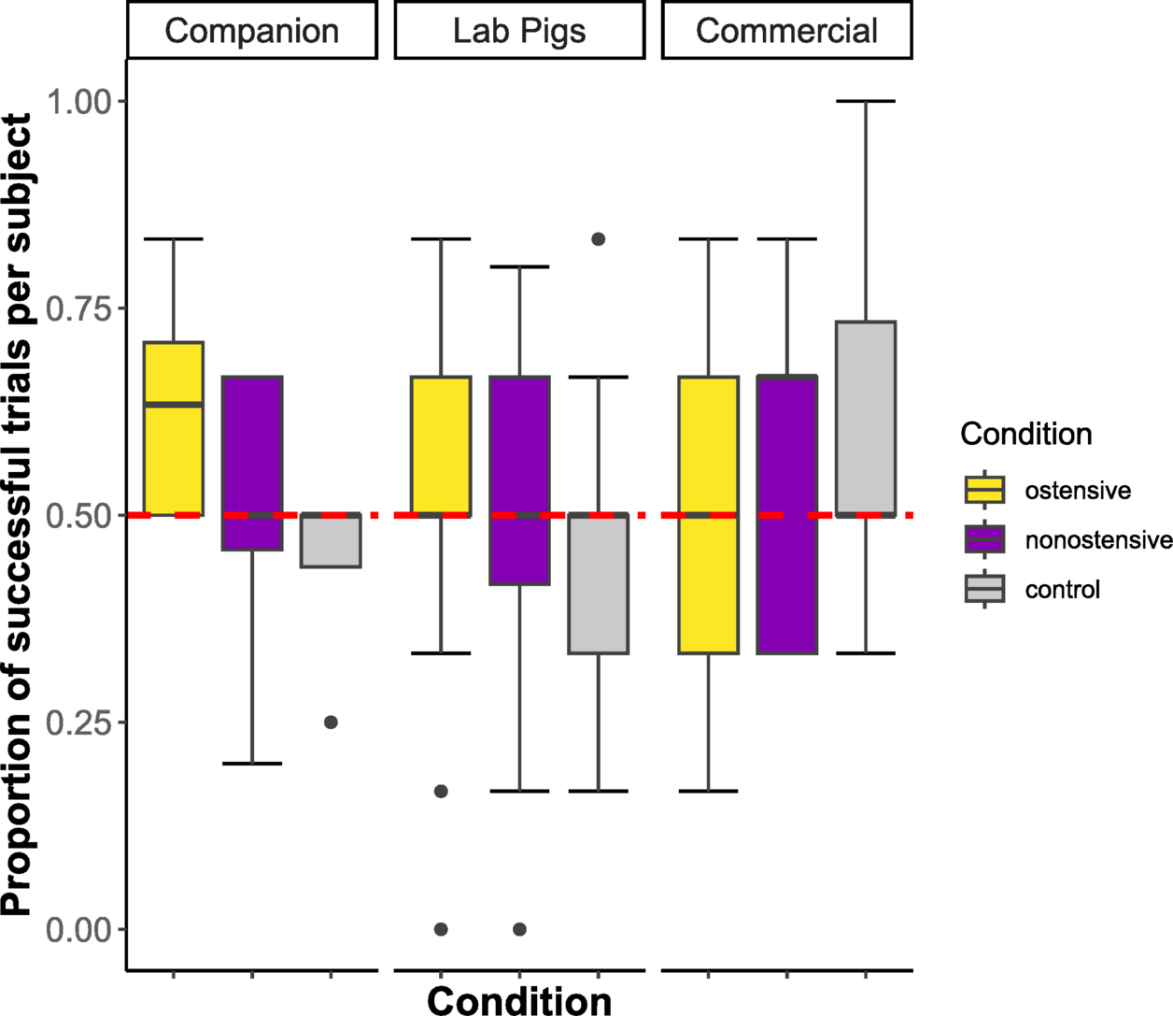
Proportion of successful trials per subject in the object-choice task for the three pig groups and three conditions (ostensive in yellow, non-ostensive in purple and control in gray).

We compared the three pig groups’ success in locating the baited one of the two bowls across an ostensive condition, a non-ostensive condition, and a control condition in which no directional cues were provided. Overall, pigs successfully followed the experimenter’s directional cues in 51% of the trials. The predictors of interest condition and pig group, or their interaction, did not significantly influence pigs’ success in following the experimenter’s directional cues in the object-choice task (full-null model comparison: *n* = 904 trials across 54 pigs, *χ²* = 5.51, *df* = 8, *p* = 0.70, see Fig. 2, for full model results see Table S12).

### Choice—A-not-B task

**Fig. 3:**
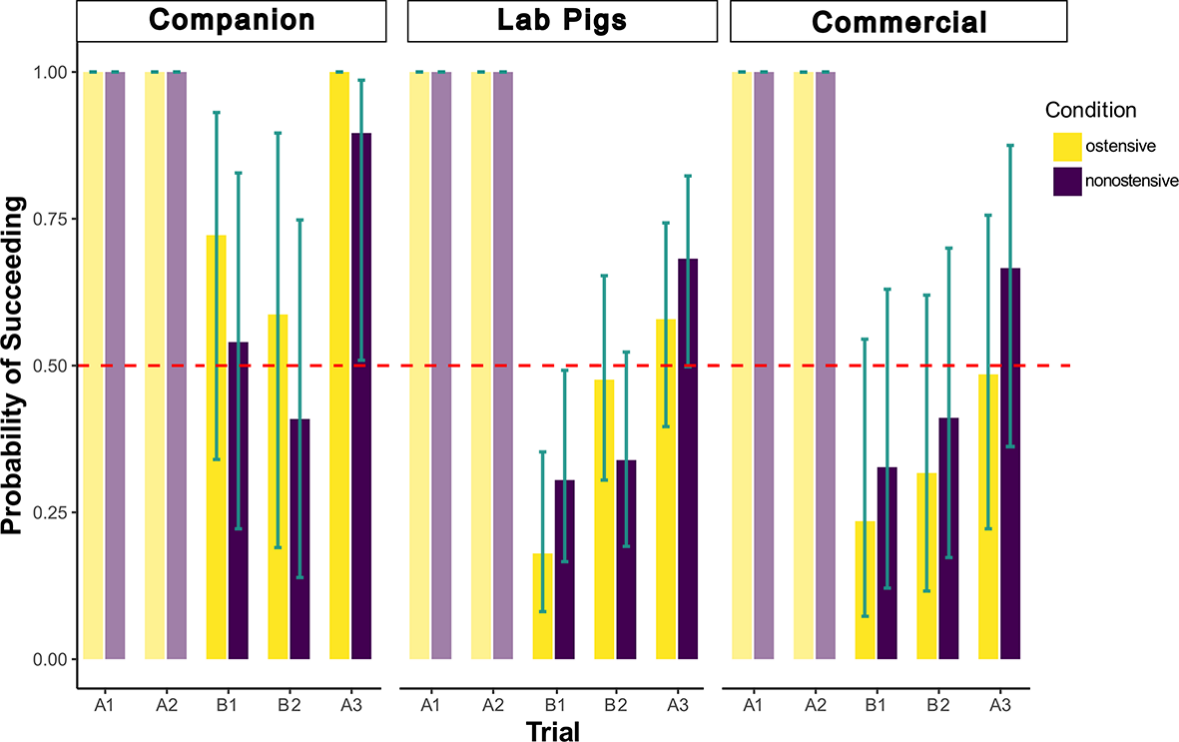
Probability of succeeding (model estimates) per trial, condition (ostensive in yellow and non-ostensive in purple) and pig group. The dashed red line indicates chance level (0.5). The turquoise bars represent the confidence intervals of the model. Trials A1 and A2 were per definition successful.

We analyzed pigs’ success in finding the food behind the correct screen in the 3rd to 5th trials (B1, B2 and A3) of the A-not-B task (for methodological details see section “Methods”). Trials A1 and A2 were by definition successful (see Methods section) and were therefore not considered in the analysis. The full model provided a significantly better fit to the data than the null model (*n* = 297 trials across 50 pigs, *χ²* = 49.46, *df* = 17, *p* < 0.001, for full model results see Table S13). Neither the interaction between condition and group (*χ²* = 2.943, *df* = 2, *p* = 0.23), nor between condition and trial (*χ²* = 2.326, *df* = 2, *p* = 0.31) was significant. We did however find significant effects of the single predictors pig group (*χ²* = 10.675, *df* = 2, *p* = 0.004) and trial (*χ²* = 328.269, *df* = 2, *p* < 0.001), while condition did not have a significant effect on the pigs’ choice (*χ²* = 0.153, *df* = 1, Fig. 3).

The highest proportion of perseverance error, i.e., lowest success, was observed in lab pigs’ B1 trials in both conditions (ostensive: 0.18 [CI: 0.08; 0.35]; non-ostensive: 0.30 [CI: 0,17; 0.49], see Table S13, see Fig. 3), performing clearly below chance level as soon as the food location had been changed, thereby indicating that lab pigs committed the A-not-B error in both conditions. In trial A3, however, lab pigs performed at chance level (ostensive: 0.58 [CI: 0.40; 0.74]; non-ostensive: 0.68 [CI: 0.50; 0.82], see Table S14). Commercial pigs performed at chance level in all trials (B1, B2 and A3) of both conditions (ostensive and non-ostensive), see Table S14.

In contrast to lab and commercial pigs, companion pigs performed significantly above chance level in both conditions of the A3 trials (ostensive: all pigs chose correctly; non-ostensive: 0.67 [CI: 0.51; 0.91], see, see Table S14), while performing at chance level in both B trials. This may indicate that their chance-level performance in the B trials was the effect of the experimenter’s hiding rather than simply the repeated reinforcement of the other location (i.e., location A before the B trials and location B before trial A3).

### Latency—detour task

**Fig. 4:**
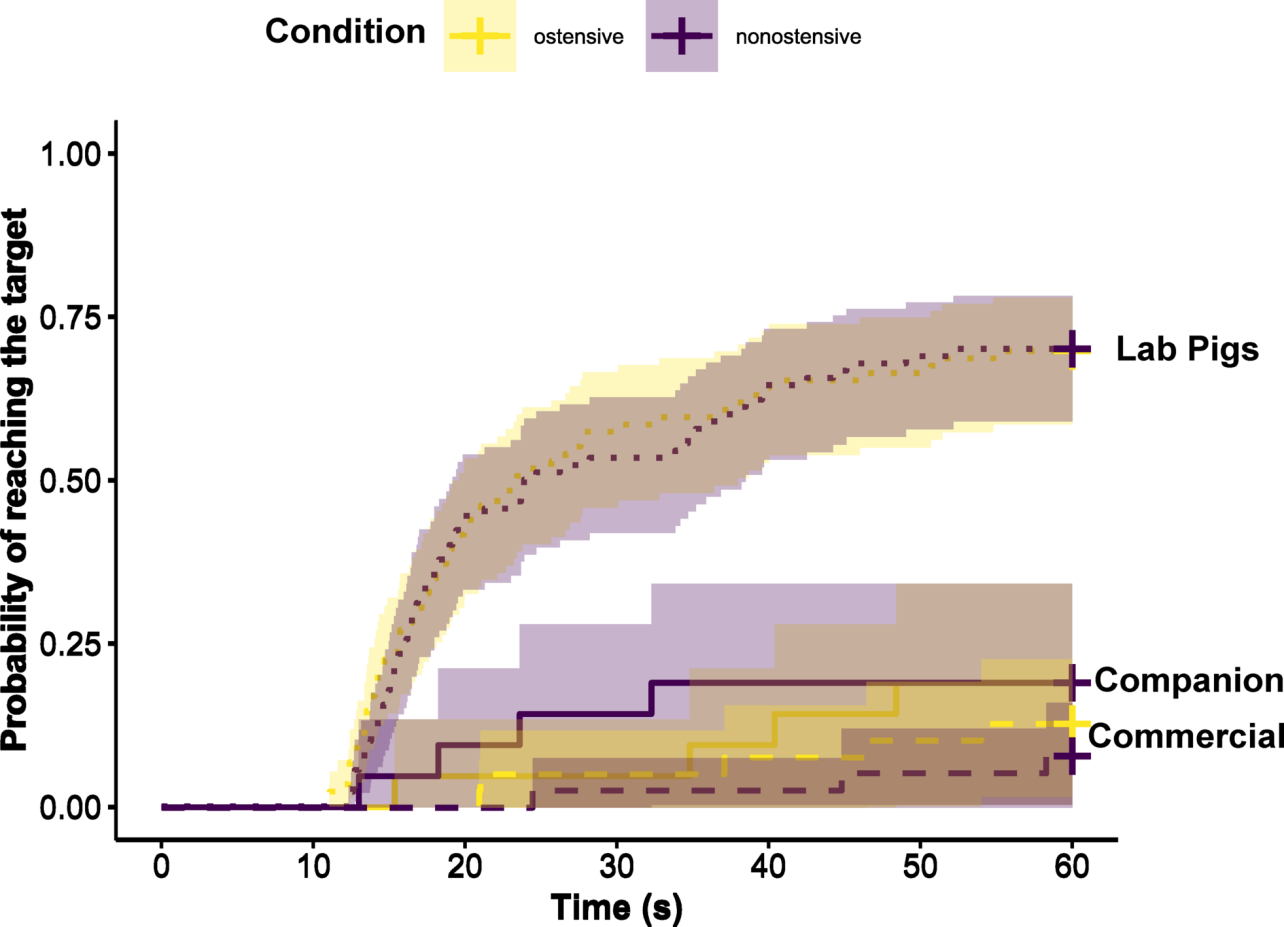
Probability of reaching the target across time (max. 60 s, i.e., the length of a trial), including the 95%-confidence intervals, for the three pig groups (Commercial = dashed line, Companion = solid line, Lab Pigs = dotted line) and two conditions (ostensive in yellow and non-ostensive in purple). Crosses at the end indicate censored datapoints, i.e., trials in which the pig did not reach the target within 60 s.

We used Cox Mixed Effects models to compare the three pig groups’ latencies to reach the target (in combination with reaching the target yes/no) in the demonstration trials (see “Methods” section) of the detour task between the ostensive condition and the non-ostensive condition. We controlled for pigs’ average latency in the no-demonstration trials. Pigs that had not reached the target by the end of the 60-second trial were assigned a latency of 60 s. The full model for the pigs’ latency to reach the target in the detour task provided a significantly better fit to the data than the null model (*n* = 298 trials across 50 pigs, *χ²* = 42.19, *df* = 11, *p* < 0.001, for full model results see Table S15). The three-way interaction did not have a significant effect (*χ²* = 0.79, *df* = 2, *p* = 0.67), and none of the two-way interactions was significant either (condition and pig group: *χ²* = 0.40, *df* = 2, *p* = 0.82; condition and trial number: *χ²* = 0.62, *df* = 1, *p* = 0.43; pig group and trial number: *χ²* = 0.26, *df* = 2, *p* = 0.88). The single predictors pig group (*χ²* = 33.22, *df* = 2, *p* < 0.001) and trial number (*χ²* = 8.78, *df* = 1, *p* < 0.001) had a significant influence, but condition did not (*χ²* = 0.01, *df* = 1, *p* = 0.94), see Fig. 4. Pairwise comparisons of the pig groups revealed that lab pigs’ latencies differed significantly from those of commercial (*z* = -4.937, *p* < 0.001) and companion (*z* = -3.305, *p* = 0.0027) pigs, but commercial and companion pigs’ latencies did not differ significantly (*z* = -2.033, *p* = 0.10). In addition, we found that the lab pigs were clearly more successful in this task than the other two groups: Three out of seven companion pigs, seven out of 13 commercial pigs and all 30 lab pigs solved the detour task within 60 s in at least one of their 12 trials.

To see whether the lack of significant difference in the latency to solve the detour task between conditions was due to the ineffectiveness of the human demonstrations in general or whether the demonstration was equally effective in both conditions, in a follow-up analysis, we compared pigs’ latencies between the demonstration trials (ostensive and non-ostensive) to a no-demonstration control condition (see Supplementary Information 4). The findings indicate that both ostensive and non-ostensive demonstrations effectively reduced at least lab pigs’ latencies relative to the no-demonstration trials (Fig. S8, Tables S16 and S17).

In 91 of these 135 successful demo trials, pigs opted for the same side the experimenter had walked on (i.e., left in the J set-up and right in the mirror-J set-up) and in 44 they chose the opposite side. Regarding the side pigs were successful on, in 244 successful trials (demo and no-demo), pigs chose to circumnavigate the fence on the left in 150 trials and on the right in 94 trials. Looking only at the 135 successful demo trials, pigs walked around the left side in 77 trials and around the right side in 58 trials.

## Discussion

In the present study, we investigated sensitivity to human ostensive communication in pigs, a domesticated species not selected for companionship or cooperation with humans, by comparing three pig groups that differ in their experience with human communication. We found that pigs, independently of their experiences, were more attentive to ostensive than non-ostensive human detour demonstrations. However, we found no similar effect of ostension on attentiveness in an A-not-B task or in an object-choice task, and pigs’ attentiveness-independent performance in none of the three tasks was enhanced by ostension. More precisely, pigs struggled to follow directional gaze and body orientation cues independently of ostension, committed the A-not-B error in both conditions and at least lab pigs were able to learn from both ostensive and non-ostensive human detour demonstrations. Even though the pig groups did not differ in their sensitivity to ostension, they did differ in their attentiveness to human demonstrations and also in their success in two out of the three tasks. That is, lab pigs were generally most and commercial pigs least attentive to human demonstrations. Noteworthily, companion pigs’ attentiveness was intermediate between commercial and lab pigs’. In addition, lab pigs performed best in the detour task and were most likely to commit the A-not-B error.

### Attentiveness—effect of ostension

Unlike for dogs^16,19,21^, horses^17^, and human infants^19^, ostension did not impact pigs’ performance in the three tasks, but it did increase their attentiveness in the detour task. Importantly, this effect of ostension persisted also when controlling for the increased length of non-ostensive demonstrations as compared to the ostensive ones (see Supplementary Information 4). We found a similar attention facilitating effect of ostension in the object-choice task; this tendency however did not persist when controlling for the length of cueing in the two conditions. Ostension did not affect attentiveness in the A-not-B task. One reason for the restricted effect of ostension could be that the detour task was the most challenging task. The difficulties pigs faced in the no-demonstration trials that preceded the demonstration trials in both conditions could have made pigs more receptive to potentially helpful ostensive human demonstrations. In contrast, in the A-not-B task, pigs were also allowed to retrieve the food if they chose incorrectly. Therefore, the only costs of not watching the demonstration closely enough were the negligible extra effort and time it takes to check the incorrect screen first. Alternatively, one could argue that, in the detour task, the demonstration was longer than the cueing in the object-choice and A-not-B tasks (on average 13.08 s), which could have created more room for variation between conditions.

### Attentiveness—differences across pig groups

In addition to the effects of ostension on the attentiveness of all pigs, we also detected considerable group differences (independently of ostension) in pigs’ attentiveness. That is, commercial pigs were generally less attentive than companion and lab pigs, with the latter being most attentive in the detour task. Multiple factors may have contributed to these differences. First, lab pigs might have been most and commercial pigs least attentive due to the age difference between groups. While lab pigs were between seven and ten years old at the time of testing, the oldest commercial pig was only three years old, due to the artificially shortened lifespan of commercial breeding sows. A recent study on horses found that older horses were more successful at following human-given cues^25^. Even though attentiveness was not explicitly measured, the authors discuss that older horses might be more attentive and less distracted than their younger peers. In addition, pigs’ inhibitory control has been shown to improve with age^26^ in a study comparing 9- and 16-week-old piglets. This suggests that their older age allowed the lab pigs to be more attentive and less distracted in our study.

Independently of age, training experience can be considered a second important driver of the observed group differences. For instance, Veit et al.^27^ suggest that the reason pigs were most attentive to a human demonstration (as opposed to a conspecific and a ghost demonstration) in a social learning study could be their training experience or their learned association between food and humans. Contrary to the lab pigs that participated in our and Veit et al.’s study, as well as contrary to the companion pigs, the commercial pigs in our sample were the only group that had had no previous testing or training experience and had seldom received food directly from humans, as they are fed via automatic feeding systems. Therefore, they are unlikely to have formed strong associations between humans and food. This, in addition to their young age and lack of experimental experiences, might have further decreased the commercial pigs’ motivation to attend to human actions.

Third, we cannot rule out that the low levels of attentiveness in the commercial pig group might be the results of the pigs’ manipulation of the fence. In some cases, the commercial pigs might have been looking at the experimenter, and potentially paid attention, despite not having been coded as “attentive”. This is because, according to the ethogram, a pig was not considered attentive when it visibly manipulated other objects, such as the door of the start box, even if its head was oriented towards the experimenter. The flexibility of the fence from which the door was built in the case of the commercial pigs might have invited manipulation of the door more than the sturdier materials used for the start boxes of the companion and lab pigs. Despite this difficulty, we made sure to carefully code attentiveness. Also, despite commercial pigs’ higher manipulation and in line with the results for the other pigs groups, commercial pigs were more attentive to ostensive than to non-ostensive detour demonstrations.

Considering all the differences between the pig groups that likely affected their human-directed attentiveness, it is even more remarkable that they all paid more attention to ostensive human detour demonstrations than to non-ostensive ones. This suggests that a limited amount of exposure to humans (the amount that commercial pigs experienced in the course of one to three years) is sufficient for pigs to develop such a sensitivity to human ostension.

### Performance and use of human-given information—effect of ostension

In contrast to their attentiveness, we did not find evidence that human ostension affects how pigs use human-given information. Our pattern of results is similar to that reported for cats which were more attentive to ostensive cues in an object-choice task, even though this increase in attention did not lead to an improvement in performance^22^. There are several potential reasons why we failed to detect an effect of ostension on pigs’ learning.

First, our results may indeed indicate that pigs, unlike dogs and human infants, do not interpret ostensive human demonstrations differently than non-ostensive ones. If so, one might conclude that neither domestication (and minimal taming) nor extensive exposure to human communication are sufficient to make animals’ learning susceptible to human ostension. Instead, selection for companionship and co-working with humans may be a crucial component that has contributed to dogs’ and horses’ sensitivity to human ostension. In contrast to these species, the selection of pigs has focused on production traits rather than communication with human caregivers^14^. Thus, for pigs, learning selectively from ostensive human demonstrations might have been of minor importance during their domestication history. Rather, adjusting to human behavior in general, regardless of whether the animals are addressed or not, might have helped pigs to obtain food and to cope with human proximity.

A second reason could be that the tasks we employed were simply not suitable for detecting an effect of human ostension on pigs’ learning. For example, detouring an obstacle might not be sufficiently ecologically relevant for pigs. However, our analyses show that, unlike for horses^28,29^, our demonstration was effective in reducing pigs’ latency, suggesting that the lack of significant difference between conditions cannot solely be attributed to a floor effect. In contrast, in the A-not-B task, the pattern of results might indeed suggest poor suitability of the task for investigating the effects of human ostension on pigs’ learning. That is, it is questionable whether the experimenter’s demonstration is what caused pigs to commit the A-not-B error—in both conditions. Instead of ostension-induced over-generalization^7^ or obedience^16^, pigs’ high persistence could account for their error. The fact that, unlike dogs and human infants^19^, pigs perseverated in both conditions is in line with the results from an unsolvable task comparing pigs and dogs. In this study, pigs continued to manipulate and unsuccessfully attempt to solve the task for significantly longer than dogs did^30^. Because of their high persistence, pigs could hence be more prone to perseverating on a previously rewarded location (side A) than other species. This would also explain lab pigs’ and commercial pigs’ chance level performance in trial A3, after the food had been hidden behind screen B twice.

The suitability of the object-choice task with directional gaze and body orientation cues to investigate how pigs process human-given information might be similarly limited. After all, previously pigs only succeeded in following human directional cues in one study^31^, but in other experiments, conducted with the companion^32^ and lab pigs (Wondrak et al., unpublished data) in our sample, the pigs performed relatively poorly. Even though we conducted warm-up trials to let the pigs experience that always only one of the two bowls was baited and that they needed to pay attention to the experimenter, the limited number of trials that could be conducted (10–20 trials) in the given time available for testing without exceeding pigs’ attention span might have been insufficient. In addition, we used a barrier in between the pig and the experimenter for safety reasons. As the use of a barrier between the subject and the experimenter is known to decrease dogs’ success in object-choice tasks^33,34^, the barrier might have caused or exacerbated the floor effect preventing us from seeing an effect of ostension on pigs’ performance.

### Performance—differences across pig groups

Even though we did not observe an effect of experience with human communication on a potential learning-enhancing effect of human ostension in pigs, we did find significant ostension-independent group differences in performance. Precisely, lab pigs were quickest in the detour task, while companion pigs were most successful, i.e., least likely to commit the A-not-B error, in the A-not-B task.

One plausible reason for these differences is that pigs’ social and physical environment influenced their performance in the tasks. While the lab pigs live in a rich social and physical environment full of stimulation and have ample space, the commercial pigs experience (limited) social contact in relatively homogenous groups and are restricted in considerably less enriched space; and the companion pigs live in enriched human households, albeit without contact to conspecifics (except for one pig). These differences in social and physical enrichment are likely to lead to differences in behavior and cognitive performance^25,35–37^.

Environmental enrichment could be particularly essential to the lab pigs’ success in the detour task^38,39^. More precisely, the lab pigs can be expected to have already encountered more obstacles that required them to take a detour than the indoor-housed commercial pigs. Likewise, the lab pigs’ social and physical environment presumably demands a high degree of behavioral flexibility, which is also crucial to success in detour tasks^40^. However, analogous arguments could be made about the companion pig group whose performance was nevertheless significantly lower than that of the lab pigs. Consequently, a combination of lab pigs’ environment, experience and age is most likely to explain their outstanding detour success.

Moreover, the lab pigs that participated in the present study already took part in a previous detour task with a different, V-shaped set-up^41^. Given that these pigs were also shown to remember a two-step problem-solving task after four years (leading experienced pigs to outperform naïve ones), even though they had not been successful in their first attempts^27^, we cannot exclude that pigs transferred their previous knowledge to the present task. This might also explain why the lab pigs were already significantly faster than companion and commercial pigs in the no-demonstration trials of the detour task.

To sum up, the lack of significant effect of human ostension on pigs’ use of information in the present study highlights the need for testing other domesticated animals not selected for companionship and for applying modified test batteries to pigs. This would help elucidate whether our null findings stem from the specific methodology applied or whether they truly reflect pigs’ capacities—and potentially those of other domesticated non-companion animals. Even more interestingly, the ostension-independent group differences in performance that became apparent in our study emphasize the role of enrichment and individual (training) experience on animals’ cognitive performance.

## Conclusion

This is the first study to probe sensitivity to human ostension, a topic predominantly studied in companion animals, in three different populations of domestic pigs. Our findings do not only provide first insights into pigs’ sensitivity to ostensive communication but also allow us to formulate recommendations for methodological refinements in future studies.

We found that, independently of their experience with human communication, ostension can enhance pigs’ attentiveness to human demonstrations. In addition, pig groups differed in their attentiveness independently of ostension, suggesting that older, more trained and more experienced individuals are more attentive to human demonstrations.

In contrast to their attentiveness, pigs’ use of human demonstrations was not affected by ostension in any task. This may indicate that selection for companionship or cooperation with humans is indispensable for animal species’ adaptation to human ostensive communication, to the extent that animals would process information provided in an ostensive manner differently to information received without having been addressed beforehand. Our results suggest that domestication for other purposes and individual experience with human communication are insufficient to evoke such a sensitivity to human ostension. Alternatively, however, it is conceivable that the experimental paradigms we employed, following previous research on human infants, dogs, cats and horses, might be less suitable for detecting such effects of ostension in pigs. As for attentiveness, we found pronounced ostension-independent group differences in pigs’ detour success and in their propensity to commit the A-not-B error. These differences illustrate the role of previous experience with humans, and with training, in modulating animals’ cognitive performance. In particular, our results highlight the importance of enrichment and age on animals’ performance in cognitive tests^42^.

Importantly, when interpreting the diverging performance of the three groups, we have to acknowledge several confounding differences between the populations. Apart from the difference that interests us, namely experience with human communication, the groups were also highly unequal in age, testing experience, social and physical environment, body size, season in which they were tested, sex ratio, neutering status and many other regards. While this impedes us from pinpointing exactly where the observed differences in ostension-independent attentiveness and performance stem from, the heterogeneity of our sample also increases the external validity of our findings^43,44^. For example, the effect of ostension on attentiveness in the detour task was independent of group, indicating that it might be robust and universal in pigs. However, it would be desirable for future studies to aim for equal and sufficiently large sample sizes, as especially the low number of companion pigs in our sample limits the strength of the conclusions we can draw about pigs intensively socialized with humans.

We conclude that, in some contexts, human ostensive communication increases pigs’ attentiveness, irrespective of their individual experience with humans. At the same time, in contrast to domesticated species selected for close cooperation with humans, ostension does not enhance pigs’ performance in the tasks we employed. Further research with a wider range of species and experimental paradigms is called for to pinpoint the evolutionary prerequisites and specific contexts that make animals sensitive to “being addressed” by humans.

## Methods

### Ethics declarations

The part of the study conducted in Austria was approved by the Ethics and Animal Welfare Committee of the University of Veterinary Medicine, Vienna in accordance with the University’s guidelines for Good Scientific Practice (ethical permit number ETK-028/02/2023). The part of the study conducted in Hungary was approved by the responsible authorities at Eötvös Loránd University, Budapest (ethical permit No. PA/EA/112-2/2021). All methods were performed in accordance with the relevant guidelines and regulations.

This purely behavioral study did not inflict any harm, suffering or any invasive procedures on the animals. During training and testing, only positive reinforcement was applied.

### Subjects

Three pig populations varying in their experience with, and exposure to, human communication acted as subjects, namely miniature pigs kept as companion animals, free-ranging Kune Kune pigs raised and kept in close contact with human caretakers for behavioral research, and commercially farmed breeding sows kept for meat production. Their characteristics, origin and keeping conditions are described below, an overview is also given in Table S2. Unlike the majority of pig cognition studies, we decided to exclusively test adult, rather than juvenile, individuals.

“**Companion pigs**”: The first group, the group most socialized with humans, consisted of eight companion pigs (4 males, 4 females, Minnesota and Minnesota mixes). Seven of them are part of a long-term project by the Department of Ethology at the Eötvös Loránd University in Budapest, Hungary^30,32^. They live as companion animals in human households since piglethood and, thus, experience intense social contact with humans every day. That is, they live in their owners’ homes (indoors and/or outdoors) and were fed by their owners three times a day. Their basic diet consisted of oat flakes and vegetables scattered on the floor, complemented with animal protein (e.g., yogurt, eggs) three times a week and occasional dietary supplements when needed. They interact with their families during every-day activities like feeding, animal care or play. In addition to their experience with humans at home, the companion pigs had already participated in previous experiments on human-pig communication, for example an object choice-task with pointing cues^32^, an out-of-reach paradigm probing human-oriented referential communication^45^ as well as an unsolvable task^30^. By the time of testing, the companion pigs were between four and five years old. The eighth pig (“Rozi”, see Table S1) was recruited from a population of seven miniature pigs kept in one household. This seven-year-old individual regularly participates in animal-assisted interventions and other events that involve contact with unfamiliar humans. Given that this pig is used to frequent travelling, it was the only one from the companion pig group not to be tested at home but in an experimental room at Eötvös Loránd University. For an overview of the subjects and their characteristics see Table S1. Before the tests, the Companion Pigs had encountered the experimenter only during the habituation visits (see section “Habituation”).

“**Lab pigs**”: The ”Lab Pig” group comprised 34 (17 females, 17 vasectomized males) free-ranging Kune Kune pigs, out of which 31 (16 females, 15 males) participated in at least one task. The remaining three were excluded and/or only used for piloting due to deafness. The pigs were between seven and ten years old at the time of testing, see Table S1. These pigs were raised for the purpose of behavioral research at the Haidlhof Research Station, Bad Vöslau, Lower Austria, and kept under semi-natural conditions, as one multi-male/multi-female sounder until October 2022^46^. Then the whole group of pigs was moved to Gut Aiderbichl, Henndorf am Wallersee, Salzburg, Austria. The 5 ha pasture at Gut Aiderbichl is equipped with two A-shaped wooden huts for shelter as well as a muddy wallow. A stable (200 m²), where drinkers, beddings with rubber mats and deep straw, and a tar-covered feeding place (50 m²) are located, offers shelter from snow in winter and sun in summer. Access to the pasture is not restricted. In winter, hay as roughage is offered *ad libitum*. The animals have always had daily contact with animal keepers (feeding and health check), and are used to the presence of researchers since they have participated in various behavioral and cognitive studies in the past. Among these are a pointing task (Wondrak et al., unpublished data), a task in which they had to attend to cues of reliable and unreliable human informants^47^, and a study on social learning including human demonstrations^27^. Further, they were already tested in a previous detour task^41^, albeit in the absence of any human demonstration. All Lab Pigs are trained to respond to their individual names when called and follow the experimenter voluntarily to the test enclosure. Also in the present study, they were always rewarded for following the experimenter’s requests (positive reinforcement only). The Lab Pigs had already encountered the experimenter 5 years prior to the study for a 1-month period and on two occasional visits after, but did not have regular contact with her.

**“Commercial Pigs”**: Finally, the third group most adequately represents the majority of domestic pigs living in human care today, as they are kept for meat production. Twenty breeding sows (one to three years old, Large White, Swiss Genetics) housed at the teaching farm Vetfarm Medau in Berndorf, Lower Austria, were included in the study, out of which 15 ultimately participated in at least one. At the time of testing, the sows were between one and three years old. According to the standard procedures at the Vetfarm, the pigs are housed in groups in indoor pens with partly slatted floors. For the duration of the study, pigs were accommodated in smaller groups of two to five individuals in partly slatted floor pens (size: 879 cm × 493 cm for larger groups or 879 cm × 245 cm for smaller groups). Pigs were automatically fed three times a day and had *ad libitum* access to water in drinkers. We conducted the tests in an adjacent pen that resembled the home pen. Pigs were kept according to the routine procedures at the Vetfarm, meaning that they were checked upon daily and received medical treatment whenever necessary. After the end of the study, the sows continued to be used for breeding and meat production purposes. Even though the breeding sows were used to the regular presence of researchers, the individuals included in this study had no or very little experience with behavioral experience as the majority of studies conducted at the VetFarm includes only juveniles. The Commercial Pigs had not interacted with the experimenter before the beginning of the study (i.e., the habituation).

All pig owners gave informed consent to their pigs’ participation in the study. Informed consent has been obtained from the handlers visible on the supplementary videos to publish their images in an online open-access publication.

### Test arena

In case of the companion pigs, tests were conducted in their owners’ gardens (for exceptions see Supplementary Information 1) on the uncovered lawn, with other objects such as furniture or toys, surrounding the test area that was at least 3.5 m × 3.5 m in size. Family members other than the owner handling the pig and assisting researchers were asked to watch quietly from a distance or leave the garden, and to ensure that other pets did not interfere with the tests. We tested the lab pigs in two different test enclosures (one for the object-choice and A-not-B tasks, approximately 300 cm × 300 cm in size, and a separate one for the detour task, approximately 600 cm × 450 cm in size) consisting of metal fences set up on their pasture. The floor and the sides of the arena were covered by green tarpaulins to minimize distraction by grass and other pigs. For the commercial pigs, tests took place in an empty indoor pen (879 cm × 245 cm) with a partly slatted floor. In all cases, pigs were tested alone, i.e., they were the only pig present in the arena, garden or experimental room/pen.

In all three tasks, pigs watched the experimenter’s demonstrations from a start box, from which they were later released into the test arena. This start box consisted of fences or wood, set up in a round or square shape, with a diameter/length of approximately 150 cm for the companion pigs, 180 cm for the lab pigs, and 200 cm for the commercial pigs.

### Habituation

To familiarize all pigs with the experimenter (the same experimenter conducted all tests with all pig groups) and the commercial and lab pigs also with the novel environment, the pigs underwent a habituation session prior to the test sessions (for details see Supplementary Information 1). In case a pig had shown fearful behavior, i.e. repeated loud vocalizations (squeals) or attempts to escape from the test enclosure (e.g., emitting distress vocalizations when next to the door or trying to forcefully open the door), the session would have been aborted immediately and the pig would have been returned to its home enclosure or pasture. However, no pig exhibited such distress signals during habituation or testing.

### General procedure

Before each trial in each task, the experimenter used food to reward the pig if it had followed the experimenter or the owner/assistant to the start box from where it could observe the experimenter’s actions. The pigs were familiarized with this start box during the warm-up trials of the first task, the object-choice task.

**Fig. 5:**
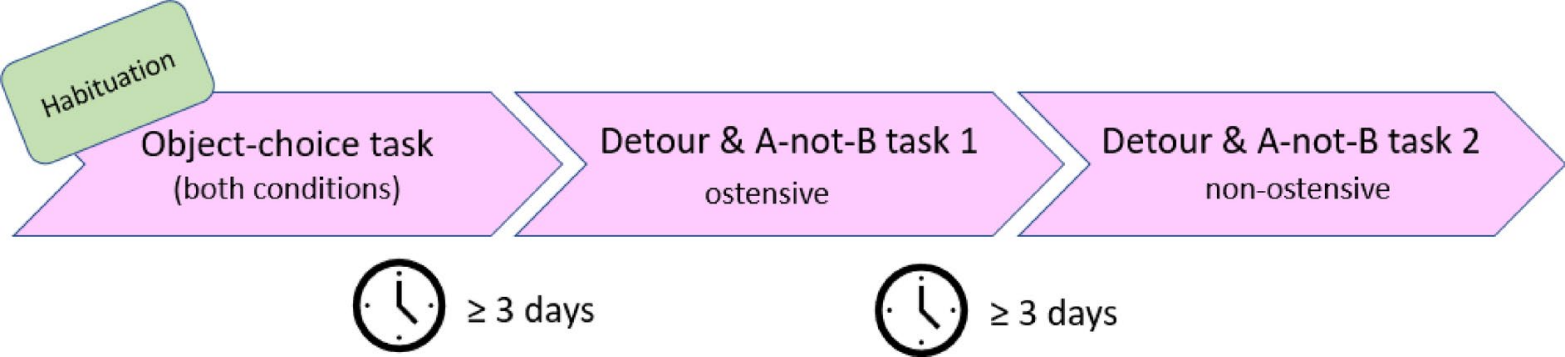
Overview of the timeline of the experiments. For some pigs, the ostensive condition of the detour and A-not-B tasks came first, for others the non-ostensive condition came first.

After the habituation, each pig underwent three test sessions, always in the same order (see Fig. 5). First, each pig completed session 1. In session 1, pigs were tested in the object-choice task, which consisted of warm-up trials, the first condition (ostensive or non-ostensive, counter-balanced across subjects), second condition (e.g., non-ostensive if the pig had seen the ostensive condition in the first block of trials), and, finally, the control trials. That is, all three conditions of the object-choice task were conducted in one single session (test day) for the object choice task. Unlike this, the A-not-B task and the detour task were spread out in two different sessions (test days). On a different day, in a second session that took place at least 3 days after the object-choice task, pigs completed the first condition (e.g., ostensive) of the detour task and the same condition of the A-not-B task. In a final session, at least 3 days after the second session, pigs completed the remaining condition (e.g., non-ostensive) of the detour task and the same condition of the A-not-B task (e.g., non-ostensive). The order in which the pigs experienced the two conditions, ostensive and non-ostensive, was counter-balanced across subjects (see Table S1 in the Supplementary Information 1).

The object-choice task was completed in one single session, which included both conditions, for two reasons. On the one hand, spreading out also the object-choice task would have required more appointments with the companion pig owners, which we visited individually, which would not have been feasible. On the other hand, the risk of carry-over effects was higher in the A-not-B task and the detour task as the solution to the problem (i.e., finding the reward behind the screen that had not been baited in the first two trials of the A-not-B task and walking around the fence in the detour task) was the same in both conditions. In the gazing task, however, there were only two possible solutions (bowls), with the correct one changing across trials in both conditions.

In addition to the order of conditions, the correspondence of the set-ups to the conditions was also counter-balanced across sexes for the lab pigs (see Table S1). The eight companion pigs were tested with a total of seven out of the eight possible combinations of condition order, condition and set-up.

To minimize the risk of carry-over effects between conditions in the A-not-B and detour tasks, we (a) conducted the three test sessions on separate days with at least 3 days in between, (b) used slightly different set-ups in the two conditions (see below) and (c) rotated the testing arena (i.e., also the starting point) by 90–180° relative to the orientation in the first condition for the companion and lab pigs. Rotating the arena was not possible for the commercial pigs due to the layout of the pen.

### Object-choice task with directional gaze and body-orientation cues

In the first task, pigs could find food in one of two food bowls if they followed the experimenter’s gaze and body orientation cues directed at the baited location.

#### Materials and set-up

**Fig. 6:**
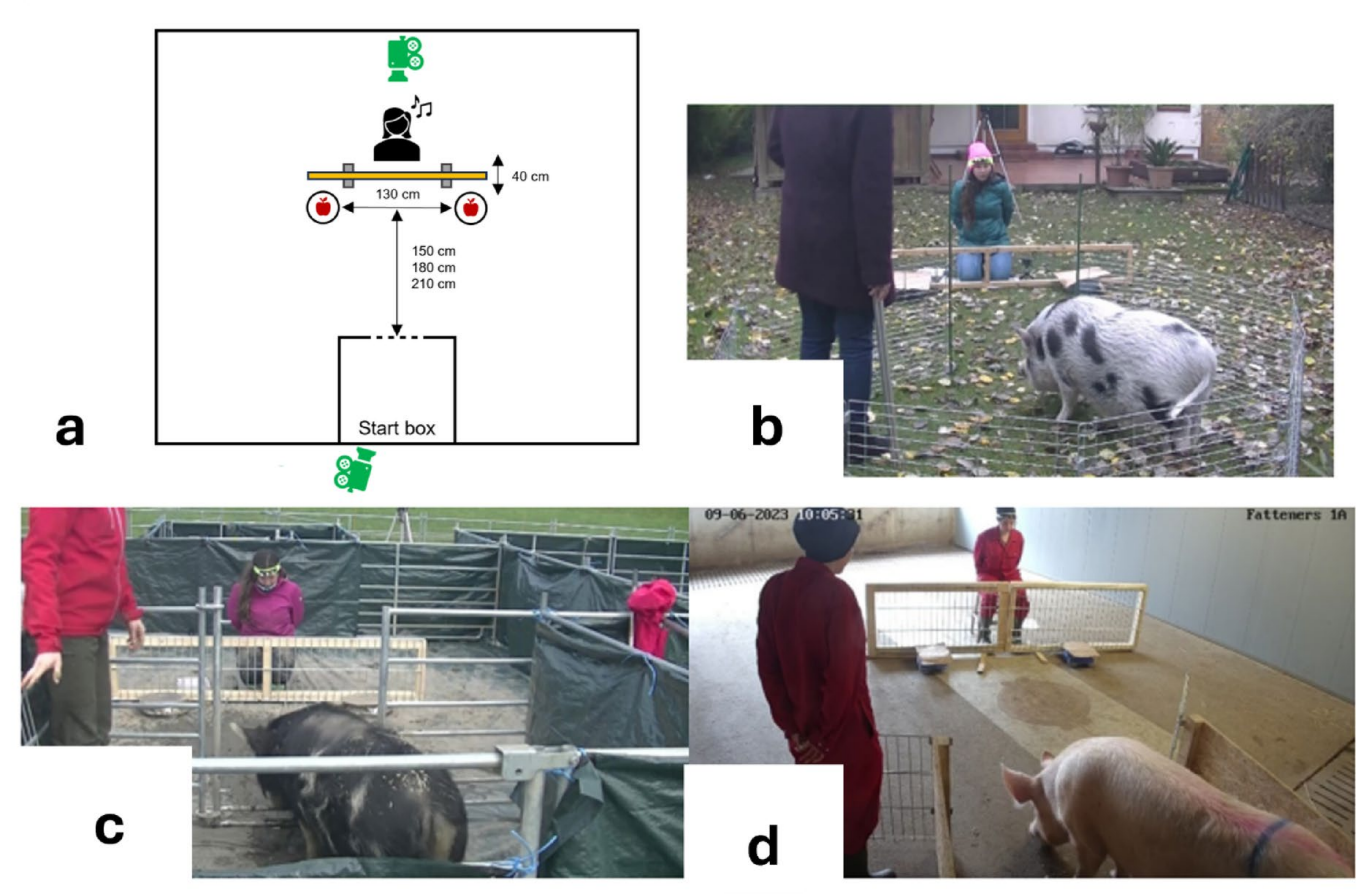
(**a**) Schematic overview of the experimental set-up in the object choice task (top view), including the position of the cameras (in green). (**b**) Picture of the set-up for the companion pigs. (**c**) Picture of the set-up for the lab pigs. (**d**) Picture of the set-up for the commercial pigs.

Two bowls were placed in front of the start box, 130 cm apart from one another^[cf. 2^. The orthogonal distance between the start box and the bowls amounted to approximately 1.5 body lengths (estimated average body length of the respective pig group; see also Supplementary Information 3 and Table S2). The experimenter was kneeling or sitting approximately 40 cm behind the imaginary midline, equidistant from both bowls^[cf. 31^. A low barrier (for details see Supplementary Information 1, section “Object-choice task”) was placed between the experimenter and the bowls (and the pig, once it had approached the bowls), see Fig. 6. During all trials, the experimenter was wearing a headband equipped with either functional or non-functional bells, depending on condition. Functional bells were used in the non-ostensive condition only, in order to control for a non-ostensive sound eliciting attention. In accordance with previous literature^24^, the bells were thus chosen as a non-ostensive, attention-getting cue to be contrasted with the clearly ostensive name-calling. The non-functional mock bells were used in the warm-up trials, ostensive trials and control trials, to allow for consistency of the experimenter’s appearance across conditions. All the sessions were recorded using two cameras. One recording from behind the start box and one from behind the experimenter.

### Test procedure

Before the test trials, pigs were made aware of the possibility that each of the bowls could contain food. For this purpose, they experienced at least ten warm-up trials where the experimenter called the pig’s attention and placed the food in one of the bowls in view of the subject, with equal numbers of trials on each side. In early warm-up trials, which were executed to establish the bowls as a food source, both bowls were presented without a cover, so that the pig could see inside and determine visually if a reward could be obtained. From warm-up trial 6 onwards and in all test trials, the bowls were covered (with cardboard or light wood) to prevent the subjects from seeing from a distance which of the bowls was baited.

In these later warm-up trials the pigs could also practice how to push the covers of both bowls to the side to reach the food in one of them. The warm-up was deemed successful if a pig approached at least one of the bowls (i.e., not necessarily the correct one first) and eventually found the food within 30 s after leaving the start box in at least eight out of the ten last trials. Once a pig passed the criterion, within a maximum of 20 trials per day on a maximum of three days, it proceeded to the test phase.

After the warm-up trials, each subject underwent 12 test trials, in two blocks of six, i.e., one block per condition (ostensive/non-ostensive). The order in which the conditions were presented was semi-randomly selected and counterbalanced across subjects (see Fig. 5). In addition, six control trials in which the experimenter did not provide any cues were conducted at the very end of the session to control for other, unintentional, cues.

The subject immediately proceeded to the first block of trials (either ostensive or non-ostensive condition) after the warm-up trials. After a break (min. 5 min), the second block of the other condition was conducted, and another break was held before the control trials. For each condition, the food was hidden in the right bowl in half of the trials and in the left one in the other half of the trials, in a semi-random order. The food was not hidden in the same bowl more than twice in a row to prevent the formation of a side bias.

Before each trial, the experimenter hid the food in one of the bowls out of the pig’s view (e.g., behind her back or while the pig was facing away from her). She then placed the bowls in front of the barrier and covered them. As soon as the bowls were in place, the experimenter attracted the subject’s attention (ostensively or non-ostensively, depending on the condition, see Supplementary Video 1).

In the ostensive trials, the experimenter established eye-contact with the subject and called the subject’s name or a familiar command. As soon as the pig looked at her, she gave three momentary, dynamic gaze and body orientation cues before the owner/assistant released the subject from the start box. The gaze cues involved a change in body orientation, i.e., not just the experimenter’s eyes and head but also her shoulders turned towards the correct food bowl. After the end of the demonstration, the owner (companion pigs) or an assistant (lab and commercial pigs) released the pigs from the start box.

In the non-ostensive trials, the experimenter pretended to play with three bells on her forehead attached to a ribbon worn around her head before each gaze cue, resulting in potentially attention-eliciting sounds and similar movements as in the ostensive condition. In between the directional cues, the experimenter did not look at the subject but instead upwards, at the bells.

In the control condition, the experimenter called the pig’s name and then remained immobile, looking down on her lap instead of at a bowl or the subject. The subject was released as soon as the experimenter had lowered her head.

A trial ended either when the subject had made a choice or 30 s after its release. After each trial, the subject was guided back to the start box either by the experimenter or by the owner/assistant. Trials in which the subject did not make a choice were repeated. Whenever a subject failed to make a choice in three consecutive trials, the test session was terminated and continued on another day.

## A-not-B task

In the second task, we assessed whether ostension would increase pigs’ attentiveness and/or enhances pigs’ tendency to commit the A-not-B error in an A-not-B task.

### Materials and set-up

The test was conducted in the same test arena as the object-choice task. As in the object-choice task, the subject was able to observe the experimenter’s actions (when she was hiding the food reward) from the start box before being released.

**Fig. 7:**
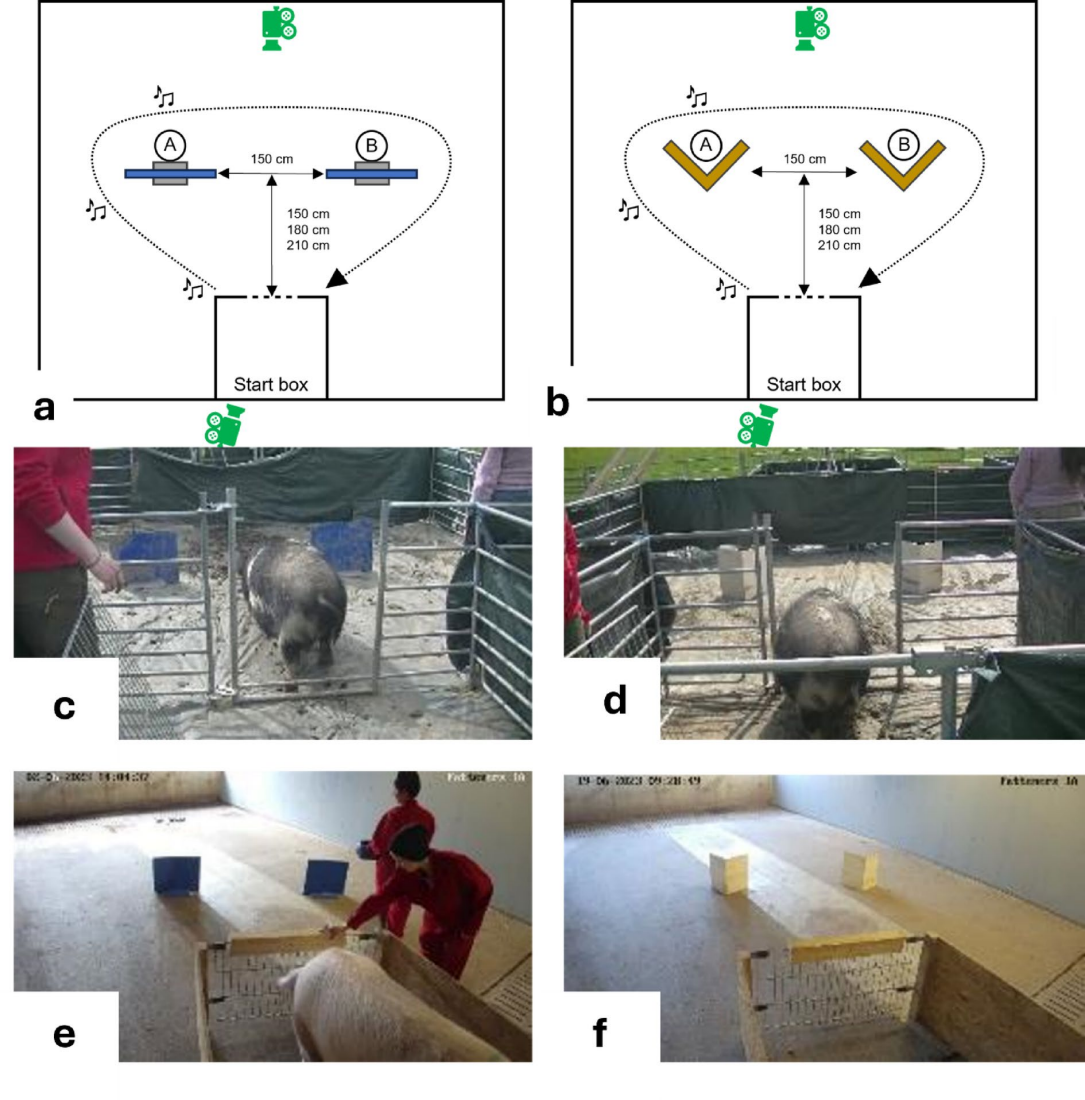
(**a**,**b**) Schematic overview of the set-up in the A-not-B task (**a**: blue plastic screens, **b**: cardboard screens) including the position of the cameras (in green). The arrow depicts the path that the experimenter took during the demonstration. The note icons along the path indicate where the three ostensive or non-ostensive utterances were given. In this example, the location A is to the subject’s left. (**c**) Example of the plastic screen set-up for a lab pig. (**d**) Example of the cardboard set-up for a lab pig. (**e**) Example of the plastic screen set-up for a commercial pig. f: Example of the cardboard set-up for a commercial pig.

To allow for each subject to be tested in both conditions (ostensive, non-ostensive) while minimizing the risk of carry-over effects, two slightly different set-ups were used. In one variant, two opaque blue plastic screens acted as potential hiding locations. Alternatively, two unfolded cardboard boxes were set up as V-shaped hiding locations. In the following, both will be referred to as “screens”. Which set-up was used in which condition was counter-balanced across subjects (see Tables S1 and S2). The screens were placed at a distance of 150 cm from one another. The distance from the start box was the same as in the object-choice task, i.e., approximately 1.5 body lengths (estimated average body length of the respective pig group), see Fig. 7. The target that the experimenter hid behind one of the screens always was a conspicuous (e.g., blue) or familiar (already associated with food) bowl baited with food.

### Test procedure

Each subject experienced both an ostensive and a non-ostensive condition on two separate testing days that were at least 3 days apart. For one of these conditions, the blue plastic screen set-up was used while the other condition was implemented using the cardboard screens. In addition, the arena was rotated by either by 90 (for some companion pigs if space in the garden was restricted) or 180° in the second session for the companion and lab pigs, relative to the spatial arrangement in the first one. Similar to the change in screen types, this rotation was performed to minimize the risk of carry-over effects. Which condition was paired with which set-up was counter-balanced across subjects, so was the side of the locations “A” (the location in which the food is hidden first) and “B” (see Tables S1 and S2). However, whether location “A” was left or right was constant across conditions for each individual subject.

Each block/condition consisted of the following sequence of trials: A–A–B–B–A (hereinafter referred to as A1, A2, B1, B2 and A3). No designated warm-up trials were conducted for this task, however, a pig only proceeded to the B trials once it had successfully approached location A first and found the food in two consecutive A trials. Otherwise, the A trials were repeated up to 30 times. After this, hiding at location B followed twice before a final A trial. The purpose of the final A trial was to assess whether pigs’ (potential) perseveration persists even after the B trials. Depending on the condition, the hiding in all trials of a block took place ostensively or non-ostensively.

Before the start of each trial, the target (i.e., the baited food bowl) was with the experimenter who was standing next to the start box. Inside the start box was the subject. In the A trials, the experimenter first crouched in front of the entrance of the start box, showed the food to the subject (including making sounds with the food/the bowl) and made the first ostensive or non-ostensive utterance. The experimenter then walked to screen A to deposit the target. On her way to location A, she uttered the second ostensive or non-ostensive attention-getter and the third one followed while she crouched and put down the bowl. She then continued her path past location B. As soon as the experimenter had reached her final position on the other side of the start box, the subject was released from the start box.

In the B trials, the experimenter repeated the same procedure, i.e., also started on the A side, passed by and sham-baited location A first (crouching and moving the food bowl behind the screen), but subsequently hid the target in location B. The number and timing of ostensive or non-ostensive attention-getters stayed the same as in the A trials, i.e., no fourth attention-getter was uttered when placing the bowl behind screen B. However, the target was moved from location A to B in a visible manner, possibly indicating the new location of the reward.

The ostensive and non-ostensive conditions differed in several regards. First, the experimenter faced the subject in the ostensive condition (i.e., turned around and established eye-contact) when she uttered the attention-getters. In contrast, she faced away from the subject (turned her back on the subject) in the non-ostensive condition^19^ and did not establish eye-contact. We expected this to create a clear contrast for the pigs, considering that pigs were shown to be able to discriminate between the front and back of human heads^48^. Second, the experimenter addressed the subject by name or used a familiar command in the ostensive condition, while she pretended to talk to herself (saying “Where could I hide the food?”) in the non-ostensive condition. The procedure of the A-not-B task is also illustrated in Supplementary Video 2.

After the subject was released from the start box, it was allowed to inspect both locations and eat the food even if it approached the incorrect location first^49,50^, in order to avoid potential conflicts with the animals. However, only the subject’s initial choice was recorded (see below). A trial ended after the subject had made a choice or 60 s after the subject’s release from the start box. The subject was guided back to the start box after each trial. Trials in which the subject did not make a choice were repeated up to three times. Testing was terminated if the subject still did not make a choice the third time.

## Detour task with human demonstrations

Inspired by Pongrácz et al.’s^21^ study on dogs, we compared pigs’ ability to learn the correct route around a detour from a human demonstrator after an ostensive and a non-ostensive demonstration.

### Materials and set-up

**Fig. 8:**
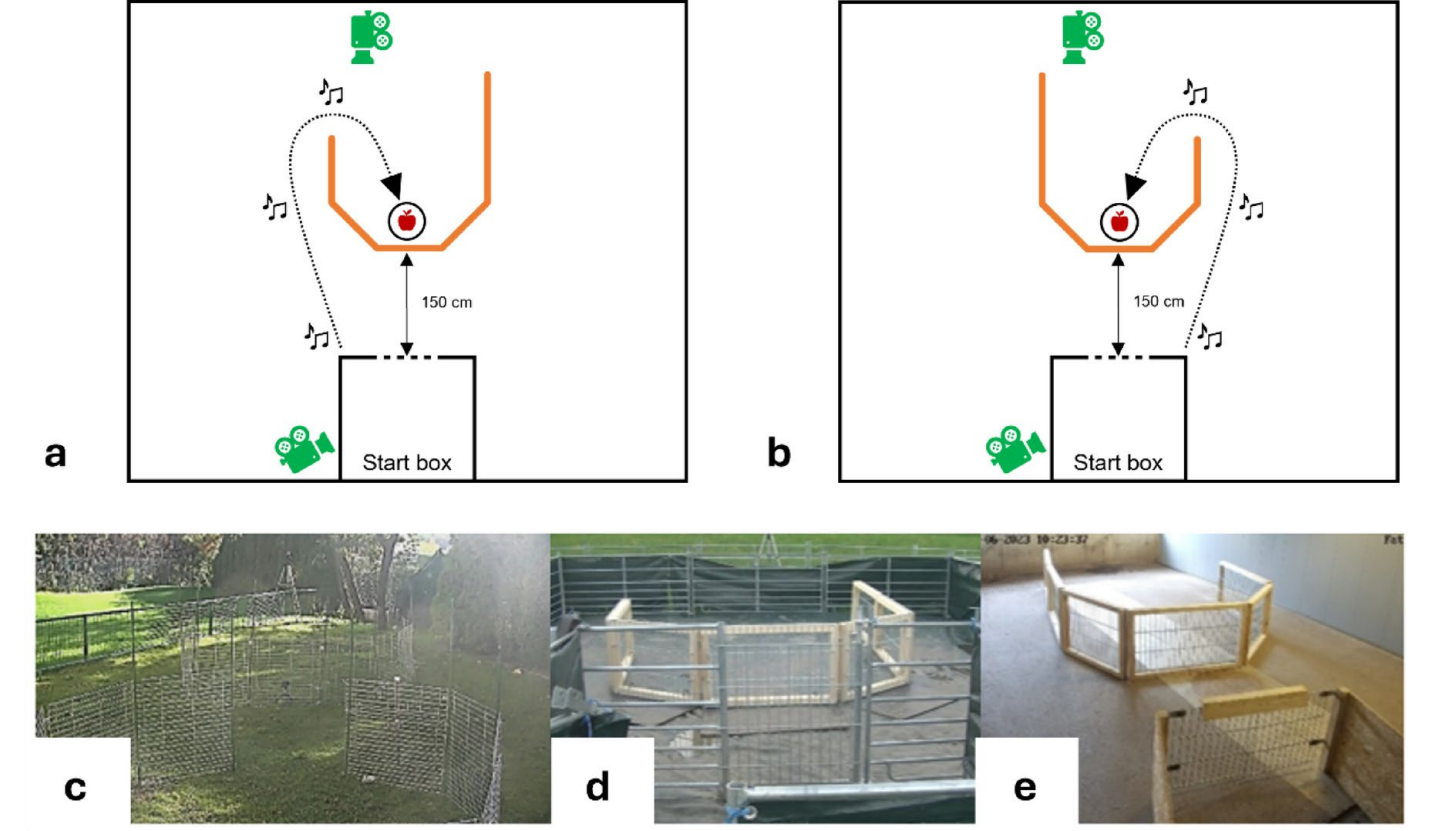
(**a**) Schematic overview of the “J” set-up in the detour task, including the position of the two cameras (in green). (**b**) Schematic overview of the “mirror-J” set-up in the detour task. c-e: Example pictures of the detour set-ups for the companion pigs (**c**), lab pigs (**d**) and commercial pigs (**e**).

To allow us to test each individual in more than one condition, the arena was rotated by 90–180° in the third session (second detour session) relative to the set-up of the second session (first detour session) for the companion pigs and two different detour set-ups were used for the two sessions/conditions for all pig groups. Both were built from portable metal fences (companion pigs) or robust metal fences with a wooden frame (lab and commercial pigs). The two different arrangements of the fence were a J-shaped inward detour and a mirror-image J-shaped inward detour (see Fig. 8). In inward detour tasks, subjects start on the outside and need to make their way to the target on the *inside* of a (form their perspective) convex fence^40^. Our reason for not exactly replicating Pongrácz et al.^21^ who used a V-shaped detour was that the lab pigs had already been tested on a V-shaped detour task in a previous study^41^, albeit without any demonstration. Thus, we used a J-shaped fence for all three pig groups instead of a V-shaped one.

Which condition, ostensive or non-ostensive, was combined with which set-up (J or mirror-image J) was counterbalanced across pigs. However, pigs whose A side was on the left in the A-not-B task experienced the mirror-image J first (for which the shorter arm, i.e., the side of the demonstration, was on the right) and vice versa (see Tables S1 and S2). For details of the set-up see Supplementary Information 1.

A familiar food bowl or, if no familiar bowl was available, a particularly conspicuous (e.g., big and blue) food bowl acted as the target. In both set-ups, it was positioned directly behind the inside of the vertex.

### Test procedure

To train the pigs to look for the target, the food bowl, we conducted one warm-up trial per condition. In these trials, the experimenter placed the food bowl in front of the fence. Therefore, the pig did not have to detour the fence yet to reach the target. The pigs were expected to directly approach the target upon being released from the start box. If a pig failed to eat the food from the bowl within 30 s, the warm-up trial could be repeated up to five times. In the event of five unsuccessful warm-up trials, the session was resumed on another day (up to three times, otherwise the pig would have been excluded), again starting with a warm-up trial. In these repeated warm-up trials, the distance between the pig and the food bowl could be decreased if needed.

In the warm-up trials, the experimenter crouched in front of the pig, showed the food in the bowl to the pig, and made sounds with the food/the bowl before placing the bowl in front of the fence. The experimenter did not talk in the warm-up trial(s).

After the warm-up trial(s), pigs immediately proceeded to the test phase. Each pig experienced a total of six test trials per condition, i.e., a sum of 12 trials on two separate days. Each block of trials consisted of three unaided “no-demonstration” trials, followed by three demonstration trials.

In the no-demonstration trials, the experimenter showed the food bowl to the pig and made sounds with it/the food before lifting it above the fence from the outside (at the vertex of the J, see Supplementary Video 3). The experimenter then reassumed her position next to the start box and the pig was released. It could then try to find its way to the target around the barrier.

In the demonstration trials, the experimenter crouched and showed the food to the pig as in the no-demonstration trials but additionally uttered the first ostensive or non-ostensive attention-getter (see Supplementary Video 3). While carrying the target around the shorter end of the fence, she either talked to the pig another two times, addressing it by name or saying a familiar command like “Come!” (ostensive condition, see ostensive attention-getters for the A-not-B task described above), or talked to herself two more times (saying “Where could I hide the food?”). The experimenter uttered the second attention-getter mid-way to the end of the short arm and the last one when she reached the end of the fence, i.e., before she entered the “inside” of the J/mirror-image J. In both cases, while talking, she turned her head and look around—either in the direction of the pig and established eye-contact (ostensive condition), or in another direction (non-ostensive condition). After putting down the bowl behind the fence and walking back on the same route, she re-assumed her position next to the start box and the pig was released.

A trial ended as soon as the subject had reached the target (i.e., touched the bowl) or after 1 min. The pig was then guided back to the start box.

If the pig (a) did not leave the start box within 30 s once, (b) left but did not approach the fence (within one head length), i.e., went away, in two consecutive trials or (c) approached the fence but did not stay in proximity (one head length) to the fence for longer than 10 s and did not return to the fence in two consecutive trials, a motivational trial (identical to the warm-up trials) was interspersed. Testing was terminated if a pig failed to retrieve the food in 5 consecutive motivational trials.

### Behavioral coding

All sessions were video recorded. The behaviors listed in Table 1 were later extracted from the videos using Loopy coding software (http://loopb.io, loopbio gmbh, Vienna, Austria).

**Table 1:** Ethogram containing the variables that were coded and analyzed in the three tasks.

Variable	Description	Task(s) for which behavior was coded
Attentiveness	Pig’s snout is oriented within the imaginary triangle between the center of its head (approximately a point between its ears) and the two bowls (object-choice task) or the two screens (A-not-B task). This is not coded if the pig is visibly occupied with something else (e.g., pushing the door of the start box, grazing) or is looking up at the owner/assistant (with its snout being higher than the horizontal).	Object-choice task, A-not-B task
Attentiveness (detour)	Pig’s snout is oriented within the imaginary triangle between its head, the vertex of the fence and 1 m on the outside next to the side of the fence detoured by the experimenter. This is not coded if the pig is visibly occupied with something else (e.g., pushing the door of the start box, grazing) or is looking up at the owner/assistant (with its snout being higher than the horizontal).	Detour task
Cueing duration	Time between the last attention-getter preceding the first of the three directional cues and the end of the last directional cue (when the experimenter has re-assumed an upright position).	Object-choice task
Demonstration duration	Time between the first cue the experimenter utters in front of the start box^1^ and the pig’s release from the start box with release being defined as the first moment the door is open wide enough so that the pig can comfortably fit through without squeezing or bending.	A-not-B task, detour task
Relative attentiveness	Attentiveness divided by the cueing duration (object-choice task) or the demonstration duration (A-not-B and detour tasks).	All tasks
Success	Pig touches the correct (i.e., baited) bowl (object-choice task) or screen (A-not-B task) before the incorrect one within 30 s upon being released.	Object-choice task, A-not-B task
Success (Detour)	Pig touches the target (bowl behind the fence) within 60 s upon being released.	Detour task
Latency to reach target	Time between the pig’s release from the start box and the moment it first touches the bowl. The maximum latency, 60 s, was assigned to pigs that had not reached the target by the end of the trial.	Detour task

^1^Note that the start of the demonstration was defined differently for the commercial pigs due to the unavailability of sound on these recordings. For this group, the start of the demonstration was defined as the last moment the bowl the experimenter was carrying touched the ground during the first utterance (in which the experimenter also moved the bowl to attract the pig’s attention).

### Inter-rater reliability analysis

A second observer independently coded 20% of the trials, proportionally split across all tasks and groups, to assess inter-rater reliability.

We calculated the inter-rater reliability for the variables relative attentiveness (all tasks), success (all tasks), detour side (detour task) and latency to reach the target (detour task) using the R package irr version 0.84.1^52^. In case of the variables success and detour side, we calculated Fleiss’ Kappa (κ), while the intraclass correlation coefficient (ICC, set to “consistency”) was calculated for the variables relative attentiveness and latency to reach the target.

Agreement was almost perfect for the variable success in the object-choice (κ = 0.966, *n* = 178) and A-not-B tasks (κ = 0.956, *n* = 100) and the two raters agreed perfectly for the success (κ = 1, *n* = 119) and side (κ = 1, *n* = 50) in the detour task. Regarding the relative attentiveness, the reliability was very good to excellent for the object-choice task (ICC = 0.871, *n* = 129), the A-not-B task (ICC = 0.898, *n* = 100) and the detour task (ICC = 0.851, *n* = 66). The raters also agreed very strongly on the latency to reach the target in the detour task (ICC = 0.996, *n* = 119).

### Statistics and reproducibility

All analyses were performed in R version 4.3.0^53^. For each of the three tasks, we analyzed the two main response variables attentiveness and performance (choice or latency). We did so by fitting a full model for each response variable in each task that contained all fixed effects of interest as well as control and random effects. In all cases, this full model was compared with a null model which lacked the main fixed effects of interest but was otherwise identical to the null model. We used a χ^2^-test (anova function, package stats version 4.3.0^53^) to compare the full model with the null model^54^. To investigate significant differences more closely, we employed the drop1 function and reduced models to test the significance of interactions and single terms^55^. To calculate pairwise differences between levels of a factor we used the functions emmeans and pairs within the emmeans package version 1.8.7^56^. The full models are reported in more detail in the following sections.

The details of the sample sizes and excluded subjects for each analysis can be found in Supplementary Information 1 and 3.

### Analysis object-choice task

We compared pigs’ attentiveness to the experimenter’s cues (i.e., the relative attentiveness calculated by dividing the time spent attentive by the duration of cueing) between the ostensive and the non-ostensive conditions of the object-choice task. We did not include data for the control condition, as attentiveness was not coded for the control condition. We fitted a Beta Regression Model (R package glmmTMB version 1.1.7^57^), setting the family argument to “ordbeta”, in order to be able to use the true bounds of the dependent variable [0,1]^58^. The main independent variable of interest was the interaction between condition (ostensive or non-ostensive) and pig group (companion pigs, lab pigs or commercial pigs). We additionally included and thereby controlled for trial number (1–6, within condition, z-transformed), condition order (whether the condition was first or second, z-transformed) and sex of the pig as fixed effects. Trial number and condition were included as random slopes within the random intercept of subject. For this purpose, condition was dummy-coded. Collinearity was not an issue as the highest variable inflation factor (VIF) was at 1.633. The model was based on 636 observations across 54 pigs.

To test the effect of the interaction between pig group and condition on pigs’ success in finding the baited bowl in the object choice task, we fitted a Generalized Linear Mixed Effects Model (R package lme4 version 1.1–33^59^) with a binomial distribution. We controlled for trial number (1–6, within condition, z-transformed), condition order (1–3, z-transformed), the side on which the food was hidden (left or right) and the pig’s sex. The pigs’ relative attentiveness could not be included as a fixed effect, given that attentiveness was not coded in the control condition. In addition, we opted for including condition order, even though this moderately increased collinearity (see below) due to the fact that the control condition always came third. Subject was considered as a random intercept with the random slopes of condition (dummy-coded) and trial number. The correlations between the random slopes and random intercept were removed as they were close to 1 or − 1^60^. The full model was not overdispersed (dispersion ratio = 1.019) and collinearity was moderate with the highest VIF being at 4.609. The model was based on 904 observations across 54 pigs.

### Analysis A-not-B task

To analyze the effect of the interaction between condition and pig group on the pigs’ attentiveness during the demonstration of the A-not-B task, we fitted a Beta regression model (R package glmmTMB version 1.1.7^57^). We controlled for trial (A1, A2, B1, B2, A3), session number (1 or 2, z-transformed), the pig’s sex, the pig’s A side (left or right), the set-up (screens or cardboard), and the number of unsuccessful A trials (“warm-up trials”) conducted before the two consecutive successful A trials (A1 and A2). The random slope of condition (dummy-coded) was included within the random intercept of subject. No collinearity was detected (all VIFs < 1.825). The model was based on 498 observations across 50 subjects.

To analyze pigs’ success in the A-not-B task, we only considered trials B1, B2 and A3, as trials A1 and A2 were per definition successful (they were repeated until the pig chose correctly in two consecutive trials). We fitted a Generalized Linear Mixed Effects Model (R package lme4 version 1.1–33^59^) with a binomial distribution. The main fixed effects of interests were condition, pig group and trial (B1, B2 or A3) as well as all possible interactions between the three. We controlled for condition order (1 or 2, z-transformed), the pig’s A side, the pig’s sex, the relative attentiveness and the set-up (cardboard or screens) as well as the number of unsuccessful A trials (“warm-up trials”). We included subject as a random effect with the random slopes of condition and set-up. The full model was not overdispersed (dispersion ratio = 1.074) and collinearity was moderate (highest VIF = 3.848). The model was based on 297 observations across 50 pigs.

To assess whether pigs performed significantly above or below chance level in trials B1, B2 and A3, we checked the 95-% confidence intervals for each combination of trial, condition and group and considered the deviation significant whenever an interval did not overlap with 0.5 (chance level).

### Analysis detour task

We fitted a Beta regression Model similar to the ones described for the object-choice and A-not-B tasks to analyze the effect of the interaction between condition and pig group on pigs’ attentiveness in the demonstration trials (trials 4 to 6) of the detour task. Additionally, session number, trial number (only demonstration trials 4–6, within condition), set-up (J or mirror-image J) and the pig’s sex were controlled for. We added condition (dummy-coded) and set-up (dummy-coded) as random slopes for the random intercept of subject. The fixed effects were found to not be collinear (all VIFs < 1.657). The model was based on 298 observations across 50 subjects.

To analyze the latency to reach the target in the demonstration trials of the detour task, we conducted a survival analysis using the Cox Mixed Effects Model (R package coxme version 2.2.18.1^61^). The response variable for such models is a combination of the time until the event occurs (in our case success, see Table 1) as well as the fact whether the event occurred (0/1). We investigated the effect of the fixed effects condition, pig group and trial number (within the demonstration trials of each condition, i.e., 4–6) as well as all possible interactions between these effects. In addition, we considered condition order, set-up (J or mirror-image-J), the relative attentiveness, the pig’s sex and the average latency in the three no-demonstration trials of the given session as control variables in the model. Collinearity between fixed effects was moderate (highest VIF = 3.500). The model was based on 298 observations across 50 subjects.

To find out whether the human demonstrations were successful in general, regardless of the condition, we fitted another Cox Mixed Effects Model (R package coxme version 2.2.18.1^61^) investigating the effect of the interaction between trial type (no-demonstration or demonstration), session number and pig group on the latency to reach the target, while controlling for trial number (within session, 1–6), set-up (J or mirror-image J) and the pig’s sex. Collinearity between fixed effects was not an issue (highest VIF = 1.784). The model was based on 598 observations across 50 subjects.

## Electronic supplementary material

Below is the link to the electronic supplementary material.

Supplementary Material 1

Supplementary Material 2

Supplementary Material 3

Supplementary Material 4

Supplementary Material 5

Supplementary Material 6

## Data Availability

Raw data are available as supplementary material.
