# Supplementary material for: Human ostension enhances attentiveness but not performance in domestic pigs
Source: Sci Rep. 2025 May 9;15:16161. doi: 10.1038/s41598-025-00511-7 (PMC12064724; doi:10.1038/s41598-025-00511-7)
Supplement: Supplementary file 4 — Supplementary Material 4 [file 41598_2025_511_MOESM4_ESM.pdf]

Supplementary Information to “Human Ostension Enhances Attentiveness but Not Performance in Domestic Pigs”

Kimberly Brosche*[1], Ariane Veit[1], Paula Pérez Fraga[2], Marianne Wondrak[3], Attila Andics[2, 4], Zsófia Virányi[1]

[1] Messerli Research Institute, University of Veterinary Medicine Vienna, Medical University of Vienna, University of Vienna, Austria

[2] Neuroethology of Communication Lab, Department of Ethology, Eötvös Loránd University, Budapest, Hungary

[3] Gut Aiderbichl, Berg 20, 5302 Henndorf am Wallersee, Austria

[4] ELTE NAP Canine Brain Research Group, Budapest, Hungary

*Corresponding author: broschekimberly@gmail.com

Messerli Research Institute, Department of Interdisciplinary Life Sciences, University Veterinary Medicine Vienna, Veterinärplatz 1, 1210 Vienna, Austria

Table of Contents

Table of Contents 1

Supplementary Information 1: Subjects and Experimental Design, Setups and Procedures.....4

Subjects 4

Companion Pigs...4

Lab Pigs...4

Commercial Pigs...5

General Procedure 6

Final Sample Size and Excluded Trials 10

Details of the Experimental Setups and Procedures...10

Test environment 10

Start box 10

Habituation 11

Object-choice task 12

A-not-B Task 14

Detour Task 15

Supplementary Information 2: Follow-up Analyses Controlling for Differences in the Experimental Set-up within the Commercial Pig Group 16

Attentiveness Object-Choice Task with Directional Gaze and Body Orientation Cues 16

Succes Object-Choice Task with Directional Gaze and Body Orientation Cues................17

Attentiveness A-not-B Task 18

Success A-not-B Task...19

Conclusions 19

Supplementary Information 3: Model Output Tables and Statistical Details 20

Attentiveness Object-Choice Task 20

Attentiveness A-not-B Task 21

Attentiveness Detour Task 22

Success Object-Choice Task 23

Success A-not-B Task....24

Latency to Reach the Target in the Detour Task Between Conditions....26

Supplementary Information 4: Follow-up Analyses of Attentiveness Controlling for Differences in Demonstration Duration....29

Object-Choice Task with Directional Gaze and Body Orientation Cues....30

A-not-B Task....31

Detour Task with Human Demonstrations....32

Conclusions....32

References Supplementary Material....33

Supplementary Information 1: Subjects and Experimental Design, Setups and Procedures

Subjects

Companion Pigs

Eight mixed-breed companion pigs, four males and four females, participated in the present study. At the time of testing, the companion pigs were between four and five years old.

Seven of the eight pigs are part of a long-term project by the Department of Ethology at the Eötvös Loránd University in Budapest, Hungary. In the course of this project, the animals were adopted by human families at approximately eight weeks of age to allow for a socialization similar to family dogs'[1,2]. These seven pigs are all housed individually, i.e., they are and have been the only pig (but not necessarily the only pet) in the household.

The eighth pig ("Rozi", see Table S1) was recruited from a population of seven miniature pigs kept in one household. This seven-year-old individual regularly participates in animal-assisted interventions and other events that involve contact with unfamiliar humans. Given that this pig is used to frequent travelling, it was the only one from the companion pig group not to be tested at home but in an experimental room at Eötvös Loránd University. For an overview of the subjects and their characteristics see Table S1 and for more details about the companion pigs see also [3].

The companion pigs had previously participated in experiments on human-pig communication, for example an object choice-task with pointing cues[1], an out-of-reach paradigm probing human-oriented referential communication[4] as well as an unsolvable task[2].

Lab Pigs

This group comprised 34 (17 females, 17 vasectomized males) free-ranging Kune Kune pigs, out of which 31 (16 females, 15 males) participated in at least one task. The remaining three were excluded and/or only used for piloting due to deafness. The pigs were between seven and ten years old at the time of testing, see Table S1.

These pigs were raised for the purpose of behavioral research at the Haidlhof Research Station, Bad Vöslau, Lower Austria, and kept under semi-natural conditions, as one multi-male/multi-female sounder until October 2022[5]. Then the whole group of pigs was moved to Gut Aiderbichl, Henndorf am Wallersee, Salzburg, Austria, where all of them will remain until their natural death. The 5 ha pasture at Gut Aiderbichl is equipped with two A-shaped wooden huts for shelter as well as a mobile drinker and a muddy wallow. A stable (200 m^2), where drinkers, beddings with rubber mats and deep straw, and a tar-covered feeding place (50 m^2) are located, offers shelter from snow in winter and sun in summer. Access to the pasture is not restricted. In winter, hay as roughage is offered *ad libitum*. The animals have always had daily contact with animal keepers (feeding and health check), and are used to the presence of researchers since they have participated in various behavioral and cognitive studies in the past. Among these are a pointing task (Wondrak et al., unpublished data), a task in which they had to attend to cues of reliable and unreliable human informants[6], and a study on social learning including human demonstrations[7]. Further, they were already tested in a previous detour task[8], albeit in the absence of any human demonstration.

All pigs are trained to respond to their individual names when called and follow the experimenter voluntarily to the test enclosure. Also in the present study, they were always rewarded for following the experimenter's requests (positive reinforcement only).

Commercial Pigs

Twenty breeding sows (one to three years old, Large White, Swiss Genetics) housed at the teaching farm Vetfarm Medau in Berndorf, Lower Austria, were included in the study, out of which 15 ultimately participated in at least one. At the time of testing, the sows were between one and three years old.

This group of pigs most adequately represents the majority of domestic pigs living in human care today, as they are kept for meat production. According to the standard procedures at the Vetfarm, the pigs are housed in groups in indoor pens with partly slatted floors. For the duration of the study, pigs were accommodated in smaller groups of two to five individuals in partly slatted floor pens (size: 879 cm × 493 cm for larger groups or 879 cm × 245 cm for smaller groups). Pigs were automatically fed three times a day and had *ad libitum* access to water in drinkers. We conducted the tests in an adjacent pen that resembled the home pen.

Pigs were kept according to the routine procedures at the Vetfarm, meaning that they were checked upon daily and received medical treatment whenever necessary. After the end of the study, the sows continued to be used for breeding and meat production purposes. Even though the breeding sows were used to the regular presence of researchers, the individuals included in this study had no or very little experience with behavioral experience as the majority of studies conducted at the VetFarm includes only juveniles.

General Procedure

As described in the main manuscript (General Procedure), the order in which the two conditions (ostensive and non-ostensive) were presented to the subjects were counterbalanced across subjects. For the A-not-B task and the detour task, each test condition was presented in a different setup. The order of the two possible setups for the A-not-B task (blue plastic screens or v-shaped cardboard screens, see Fig. S2) and the detour task (J-shaped fence or mirror-image J-shaped fence, see Fig. S3) was additionally counterbalanced across subjects. For a general overview of the counterbalancing see Table S1 and for detailed information on each test subject including the test conditions they experienced see Table S2. For more details on the experimental setups, see sections below.

Table S1: Overview of the experimental design showing the counterbalancing of conditions and set-ups across eight example subjects. Per task, the order of ostensive (O) and non-ostensive (N) condition is given (the control condition of the object-choice task is not included here as it always came third). In addition, for the A-not-B, the order of the setup (P – plastic, C – cardboard) and the side of the "A" location (L – left, R – right) are given. Similarly, for the detour task the order of the setup (J – J-shaped, MJ – mirror-image J-shaped) is given.

Task / Subject	Object-choice	A-not-B			Detour	
	Condition Order	Condition Order	Setup Order	„A" side	Condition Order	Setup Order
1	O N	N O	P C	L	N O	MJ J
2	N O	O N	P C	R	O N	J MJ
3	O N	N O	C P	R	N O	J MJ
4	N O	O N	C P	L	O N	MJ J
5	O N	N O	P C	R	N O	J MJ
6	N O	O N	P C	L	O N	MJ J
7	O N	N O	C P	L	N O	MJ J
8	N O	O N	C P	R	O N	J MJ

*Table S1: Overview of all pigs that participated in the study. Per subject, the name and group it belongs to is given, as well as the sex (M – male, F - female), date of birth, and the age at the time of testing (in years). Additionally, the order in which the ostensive (O) and non-ostensive (N) condition was presented is given per task, including the setup order for the A-not-B task and the detour task (see Table S1 for more information on abbreviations). For the A-not-B task, the number of unsuccessful A trials is given per subject and session to indicate differences in learning speed. Cells with gray shading indicate that the subject never completed this test and/or that the tests were not analyzed. Length of the barrier/distance, only in the gazing and the A-not-B task, refers to commercial pigs with short barriers being indicated by * next to the subject's name.*

Subject Information					Condition and Setup Order						Unsuccessful A trials	
**Name**	**Group**	**Sex**	**Date of birth**	**Age (in years)**	**Object-Choice**	**A-not-B**			**Detour**		**Session 1**	**Session 2**
Meaty	companion	M	05.04.2018	4	O N	N O	P C	L	N O	MJ J	1	2
Borsó	companion	M	14.08.2017	5	O N	N O	C P	R	N O	J MJ	5	1
Pilo	companion	M	14.09.2017	5	N O	O N	C P	L	O N	MJ J	0	0
Rozi	companion	F	17.07.2015	7	O N	N O	P C	R	N O	J MJ	2	3
Sári	companion	F	25.04.2018	4	N O	O N	P C	L	O N	MJ J	6	5
Lajos	companion	M	23.10.2017	4	O N	N O	C P	L	N O	MJ J	2	3
Bambi	companion	F	27.10.2017	4	O N	N O	C P	L	N O	MJ J	4	25
Töpi	companion	F	29.09.2017	4	N O	O N	P C	R	O N	J MJ	8	2
Beauty	lab	F	06.03.2013	10	N O	O N	C P	L	O N	MJ J	1	3
Zeus	lab	M	20.06.2015	7.5	O N	N O	P C	L	N O	MJ J	5	0
Raya	lab	F	22.06.2015	7.5	N O	O N	P C	R	O N	J MJ	0	2
Radomir	lab	M	22.06.2015	7.5	O N	N O	C P	R	N O	J MJ	0	4
Bibi	lab	F	26.09.2014	8.5	N O	O N	P C	L	O N	MJ J	2	3
Zampano	lab	M	28.08.2014	8.5	O N	N O	C P	L	N O	MJ J	4	0
Zoe	lab	F	28.08.2014	8.5	N O	O N	C P	R	O N	J MJ	0	5
Barbarossa	lab	M	20.06.2015	7.5	O N	N O	P C	R	N O	J MJ	0	0
Zoltan	lab	M	20.06.2015	7.5	N O	O N	C P	L	O N	MJ J	1	9
Belana	lab	F	20.06.2015	7.5	O N	N O	P C	L	N O	MJ J	1	2
Rasputin	lab	M	30.08.2014	8.5	N O	O N	P C	R	O N	J MJ	2	0
Rosine	lab	F	22.06.2015	7.5	O N	N O	C P	R	N O	J MJ	1	5
Zardoz	lab	M	20.06.2015	7.5	N O	O N	P C	L	O N	MJ J	1	0
Bijou	lab	F	26.09.2014	8.5	O N	N O	C P	L	N O	MJ J	3	0

Benjamin	lab	M	26.09.2014	8.5	N O	O N	C P	R	O N	J MJ	2	3
Zwetschge	lab	F	28.08.2014	8.5	O N	N O	P C	R	N O	J MJ	0	1
Radieschen	lab	F	22.06.2015	7.5	N O	O N	C P	L	O N	MJ J	3	3
Romeo	lab	M	30.08.2014	8.5	O N	N O	P C	L	N O	MJ J	5	1
Bella	lab	F	26.09.2014	8.5	N O	O N	P C	R	O N	J MJ	NA	NA
Zazou	lab	M	28.08.2014	8.5	O N	N O	C P	R	N O	J MJ	4	0
Rapunzel	lab	F	30.08.2014	8.5	N O	O N	P C	L	O N	MJ J	2	2
Bolero	lab	M	20.06.2015	7.5	O N	N O	C P	L	N O	MJ J	5	2
Blossom	lab	F	20.06.2015	7.5	N O	O N	C P	R	O N	J MJ	0	1
Rudi	lab	M	30.08.2014	8.5	O N	N O	P C	R	N O	J MJ	1	0
Zafira	lab	F	28.08.2014	8.5	O N	N O	P C	L	N O	MJ J	4	1
Zeppelin	lab	M	20.06.2015	7.5	N O	O N	P C	R	O N	J MJ	0	1
Bessy	lab	F	26.09.2014	8.5	O N	N O	C P	R	N O	J MJ	0	0
Bruno	lab	M	20.06.2015	7.5	N O	O N	P C	L	O N	MJ J	0	0
Zita	lab	F	20.06.2015	7.5	O N	N O	C P	L	N O	MJ J	0	7
Zacharias	lab	M	28.08.2014	8.5	N O	O N	C P	R	O N	J MJ	1	1
Blume	lab	F	26.09.2014	8.5	O N	N O	P C	R	N O	J MJ	0	0
Sow768 *	commercial	F	03.10.2020	2.5	O N	N O	P C	L	N O	MJ J	2	0
Sow847 *	commercial	F		1.5	N O	O N	P C	R	O N	J MJ	0	0
Sow842 *	commercial	F		1.5	O N	N O	C P	R	N O	J MJ	0	0
Sow833 *	commercial	F	02.10.2021	1.5	N O	O N	C P	L	O N	MJ J	0	3
Sow805 *	commercial	F		2	O N	N O	P C	R	N O	J MJ	2	0
Sow801 *	commercial	F		2	N O	O N	P C	L	O N	MJ J	0	1
Sow832	commercial	F	02.10.2021	1.5	O N	N O	C P	L	N O	MJ J	0	1
Sow841	commercial	F	02.10.2021	1.5	N O	O N	C P	R	O N	J MJ	0	0
Sow794	commercial	F	08.05.2021	2	O N	N O	P C	L	N O	MJ J	1	0
Sow797(noTag)	commercial	F	07.05.2021	2	N O	O N	P C	R	O N	J MJ	6	0
Sow775	commercial	F	09.11.2020	2.5	O N	N O	C P	R	N O	J MJ	NA	NA
Sow897/879	commercial	F	25.03.2022	1	O N	N O	P C	R	N O	J MJ	2	0

Flora	commercial	F	15.05.2022	1	N O	O N	P C	L	O N	MJ J	1	NA
Sow878	commercial	F	25.03.2022	1	O N	N O	C P	L	N O	MJ J	0	2
Sow728	commercial	F	19.01.2020	3	N O	O N	C P	R	O N	J MJ	NA	NA

Final Sample Size and Excluded Trials

A total of 54 pigs passed the warm-up phase of the object-choice task and participated in this task. Out of 972 planned trials, 902 valid trials were included in the analysis. In addition to 63 control trials, one ostensive trial and six non-ostensive trials were not conducted due to the pigs' lack of motivation.

In the A-not-B task, four pigs did not complete all trials: one lab pig could not participate at all due to lameness (Bella, Table S2), one commercial pig could not be motivated to complete the test and two more commercial pigs only completed one A-not-B test session (Flora, Sow775, and Sow728, Table 2S), in one case due to a lack of food motivation and in another case due to experimenter error leading to the same condition being presented in both sessions. This left us with a sample size of 50 pigs for the A-not-B task. Out of 500 planned trials (5 trials in each of two conditions for each of 50 pigs), 498 were conducted and were included in the analysis investigating attentiveness. In one A3 trial, attentiveness could not be coded due to equipment failure and another one was omitted due to experimenter error. When analyzing pigs' success, we only considered trials B1, B2 and A3 (see below), which comprised 297 out of 300 planned trials. In addition to the trial that was omitted, in two trials, the pig failed to choose even when we repeated this trial.

The sample size for the detour task amounted to 50 pigs. One companion pig showed fear of the fence and was therefore excluded (Rozi, Table S2), one lab pig did not participate due to lameness (Bella, Table S2), one commercial pig lacked food motivation and another commercial pig erroneously experienced the same condition twice and was therefore excluded from the analysis. Out of 300 planned demonstration trials, 298 were included in the analyses of attentiveness and latency. One trial was omitted due to experimenter error and another trial was excluded because the pig managed to obtain the food by lifting the fence over the bowl rather than by detouring the fence.

Details of the Experimental Setups and Procedures

Test Environment

As outlined in the main manuscript, the pigs were tested in three different environments. That is, the companion pigs were tested in their owners' garden (with the exception of "Rozi", who was tested in an experimental room at the university), the lab pigs were tested in a fenced-off, tarpaulin-covered enclosure on their pasture, and the commercial pigs were tested in an indoor enclosure. In addition, the set-ups slightly differed in terms of dimensions and nature of equipment used, see descriptions below.

Start Box

The start-box did not only differ in size between the groups, but they were also built from different materials (see below). To keep the pig in the start box during the demonstration and to subsequently release it, either the pig's owner (for most companion pigs) or an assistant stood inside (companion pigs) or next to (lab and commercial pigs) the start box during the tests. After each trial, the owner/assistant and/or the experimenter lured the pig back to the start box using a food reward.

In the case of the companion pigs, whom the experimenter visited in their gardens, the fences used for the detour and the start box needed to be transportable. Therefore, both the start box and the detour were built of light metal fences consisting of 60 cm × 60 cm elements that could flexibly be attached to one another. The "door" of the start box consisted either of a metal bar or a loose fence element that the owner held in place in front of the entrance of the start box, in front of the pig, to keep the pig inside, see Figure S1b, c, and d; as well as Supplementary Videos 1-3. For some companion pig individuals that were reluctant to enter the confined space of the start box, the back of the start box remained open. This, however, should not have impacted pigs' performance as they

watched the experimenter from the front of the start box and the experimenter only started the demonstration once they were watching from there. Trials in which a pig left the start box prematurely or left it via the back, rather than the front door, were considered invalid and were repeated.

For the lab pigs, the start box was built of the same material as the entire arena (metal fences, “Patura Steckfix Horden”, PATURA KG, Mainblick 1, D-63925 Laudenbach) and was directly adjacent to the main arena. The floor and the sides, except for the front of the start box through which pigs observed the experimenter, were covered in green tarpaulins, see Figure S1c, S2c/d and S3c, as well as Supplementary Videos 1-3. The door of the start box consisted of a transparent metal grid (60 cm × 70 cm) which the assistant held in place during the demonstration and lifted at the end of the demonstration to release the pig.

The commercial pigs started each trial from a wooden start box that was placed in a corner of the enclosure. The back of the start box was open towards the door of the enclosure so that pigs could comfortably enter despite their size and the assistant could subsequently close the door of the enclosure behind them, see Figure S1d, S2e/f and S3d, as well as Supplementary Videos 1-3. The front consisted of transparent metal grids that could be opened like a swinging door and locked in place using a wooden rail that the assistant could fit onto the grid when the door was closed. However, especially the lower grids remained flexible enough to give in to pigs’ manipulation with their snout.

Habituation

In the case of the companion pigs, the experimenter visited the pigs at home at least once on a separate occasion before the first test. In the presence of the owner, the experimenter invited the pigs for positive interactions (such as gentle stroking). For pigs that showed fearful or aggressive behavior towards the experimenter, such habituation visits were repeated until the pig ceased to show signs of aggression or distress (beyond the level reported as typical by the owners) in the presence of the experimenter.

For the lab and commercial pigs, the habituation phase consisted of 10-min sessions in the test enclosure. The pigs were individually guided to the test enclosure by the experimenter (using a food reward if necessary). First, they were allowed to explore the enclosure on their own for one minute. After one minute, the experimenter entered and offered positive interactions (e.g., gentle stroking) to the pigs by allowing them to approach her voluntarily. After another minute, the experimenter tried to approach (if the pig had not already approached her in the previous phase) and pet the pig during the subsequent 6 min. In the last two minutes, pigs again had the opportunity to explore the enclosure on their own.

We conducted three such habituation sessions on three separate (not necessarily consecutive) days with the commercial pigs. Due to their experience with close human interaction and behavioral testing, the lab pigs only underwent one habituation session.

Object-Choice Task

Barrier

In the object-choice task, a barrier was placed between the experimenter and the bowls (and the pig, once it had approached the bowls) to provide security to both the experimenter and the pigs. The height of the barrier and, consequently, the height at which the experimenter was sitting/kneeling differed across the pig groups due to their size difference. The barrier was approximately 30 cm high for the companion pigs, 40 cm high for the lab pigs and 60 cm high for the commercial pigs (see Figure S1 and Supplementary Video 1). The experimenter was kneeling behind the barrier on the ground (for the companion pigs) on a 20-cm high children's car seat (for the lab pigs) or sitting on a 50-cm high stool (for the commercial pigs), in a way that approximately the same proportion of her body was visible to the pigs (i.e., not partially covered by the barrier), in all cases. Note that the barrier of the commercial pigs was originally shorter (approximately 130 cm) and was lengthened (to approximately 200 cm) after the first few subjects (see Table S2). Similarly, the orthogonal distance between the start box and the bowls amounted to approximately 1.5 body lengths (estimated average body length of the respective pig group) in the object-choice test and the A-not-B test but was again shorter for the first few commercial pigs. For an overview of these exceptions see Table S1 and for an analysis of the potential influence of these changes see Supplementary Information 2.

Details of the Procedure

One of the bowls (approximately 30 cm in diameter) contained a reward (e.g., apple pieces or carrots, depending on the owner-reported preferences of the pig). Each of these bowls had a double bottom hiding additional food (equal amounts on the two sides) in order to control for olfactory cues. In addition, both bowls were rubbed in food scent prior to the session.

Before the test sessions, we conducted warm-up trials. Before each warm-up trial, the experimenter or the owner/assistant lured the subject to the start box. The experimenter then called the subject's name or a familiar command to attract its attention and showed the food reward before openly placing it into one of the bowls as soon as the subject looked at her. The sequence of trials was semi-random in a way that the food was not hidden on the same side in more than two consecutive trials. In the first five trials, the bowls were not covered to gradually familiarize pigs with the task. From trial 6 onwards, the covers were also placed on the bowls so that the subject could learn how to remove them to access the reward. After the baiting, the subject was released and was free to investigate both food bowls. The warm-up was deemed successful if a pig approached at least one of the bowls (i.e., not necessarily the correct one first) and eventually found the food within 30 s after leaving the start box in at least eight out of the ten last trials. Once a pig passed the criterion, it proceeded to the test phase. If a pig did not reach the success criterion after 20 warm-up trials, we terminated the session and started anew with the first warm-up trial on another day. If three warm-up sessions were unsuccessful due to the pig not reaching the criterion, we excluded this pig from the study.

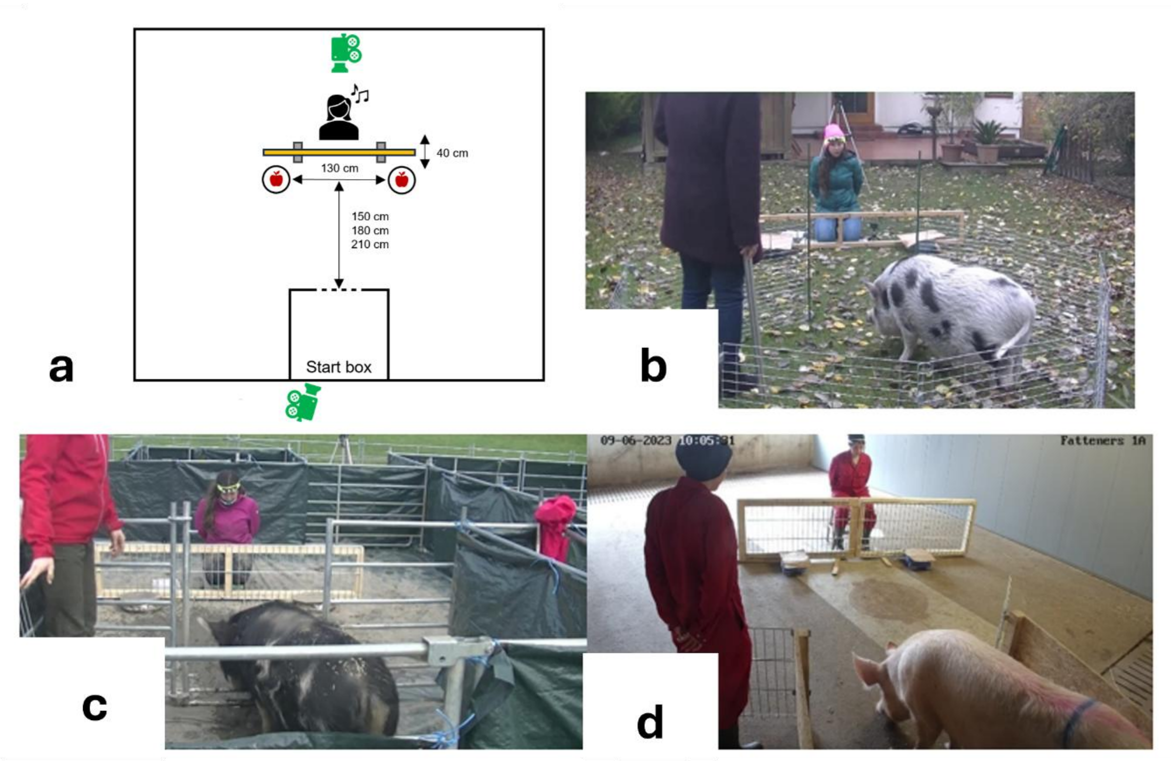

Figure S1: a: Schematic overview of the experimental set-up in the object choice task (top view), including the position of the cameras (in green). b: Picture of the set-up for the companion pigs. c: Picture of the set-up for the lab pigs. d: Picture of the set-up for the commercial pigs.

A-not-B Task

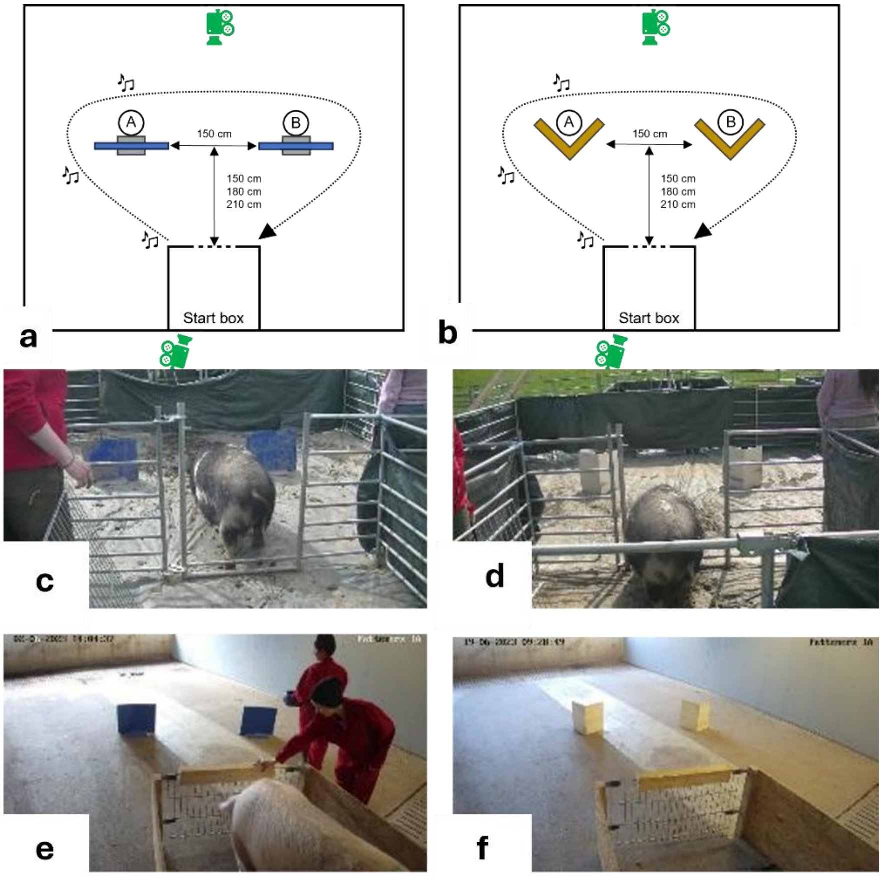

Figure S2: a and b: Schematic overview of the set-up in the A-not-B task (a: blue plastic screens, b: cardboard screens) including the position of the cameras (in green). The arrow depicts the path that the experimenter took during the demonstration. The note icons along the path indicate where the three ostensive or non-ostensive utterances were given. In this example, the location A is to the subject's left. c: Example of the plastic screen set-up for a lab pig. d: Example of the cardboard set-up for a lab pig. e: Example of the plastic screen set-up for a commercial pig. f: Example of the cardboard set-up for a commercial pig.

Detour Task

The total length of the fence in the detour task was approximately 600 cm for the companion pigs, 610 cm for the lab pigs and 735 cm for the commercial pigs. The vertex of the J was approximately 150 cm away from the start box for all pig groups. The length of the longer arm of the J (measured from the inside of the vertex to the imaginary line perpendicular to the end of the long arm) amounted to 42% of the overall fence length, the length of the shorter arm amounted to 28% of the overall fence length. The width of the fence was 41% of the entire fence length. The fence was approximately 60 cm high in all cases.

Given pigs' strong natural propensity to root and lift objects with their snout [9], the fence was fixed in place for the companion and lab pigs by anchoring it in the ground. This was not possible in the indoor arena in which the commercial pigs were tested. For visualizations see Figure S3 and Supplementary Video 3.

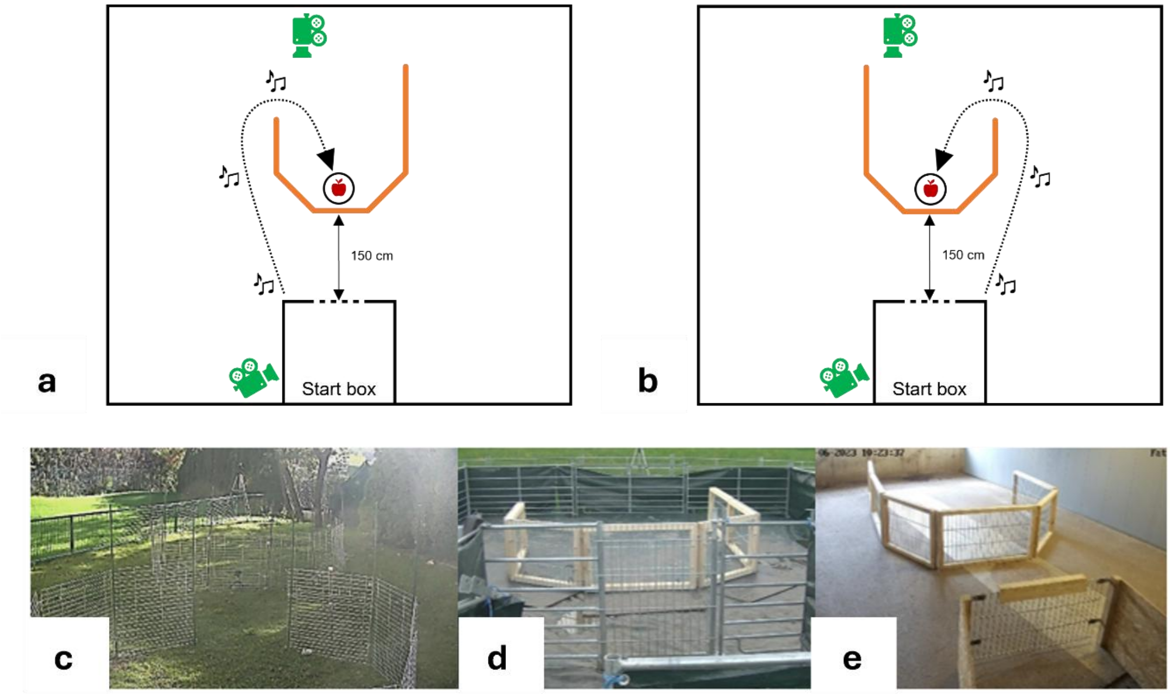

Figure S3: a: Schematic overview of the "J" set-up in the detour task, including the position of the two cameras (in green). b: Schematic overview of the "mirror-J" set-up in the detour task. The arrow depicts the path that the experimenter took during the demonstration. The note icons along the path indicate where the three ostensive or non-ostensive utterances were given. c-e: Example pictures of the detour set-ups for the companion pigs (c), lab pigs (d) and commercial pigs (e).

Supplementary Information 2: Follow-up Analyses Controlling for Differences in the Experimental Set-up within the Commercial Pig Group

Given that the distance between the start box and the bowls (object-choice task) or screens (A-not-B task) was changed midway through the testing for the commercial pigs, we here descriptively compare attentiveness and success between individuals that experienced the short and those that experienced the long distance.

Attentiveness Object-Choice Task with Directional Gaze and Body Orientation Cues

Pigs' attentiveness in the object-choice task for the two groups is plotted in Figure S4. As can be seen from the plot, pigs that were tested with the shorter distance and barrier (i.e., those for which the lengths deviated from the proportion used for the other populations) were more attentive but did not clearly differentiate between conditions, counter to what is reflected in the overall results for the commercial pigs (see main manuscript). Therefore, this sub-sample is unlikely to have generated false positive results regarding differences between conditions and groups.

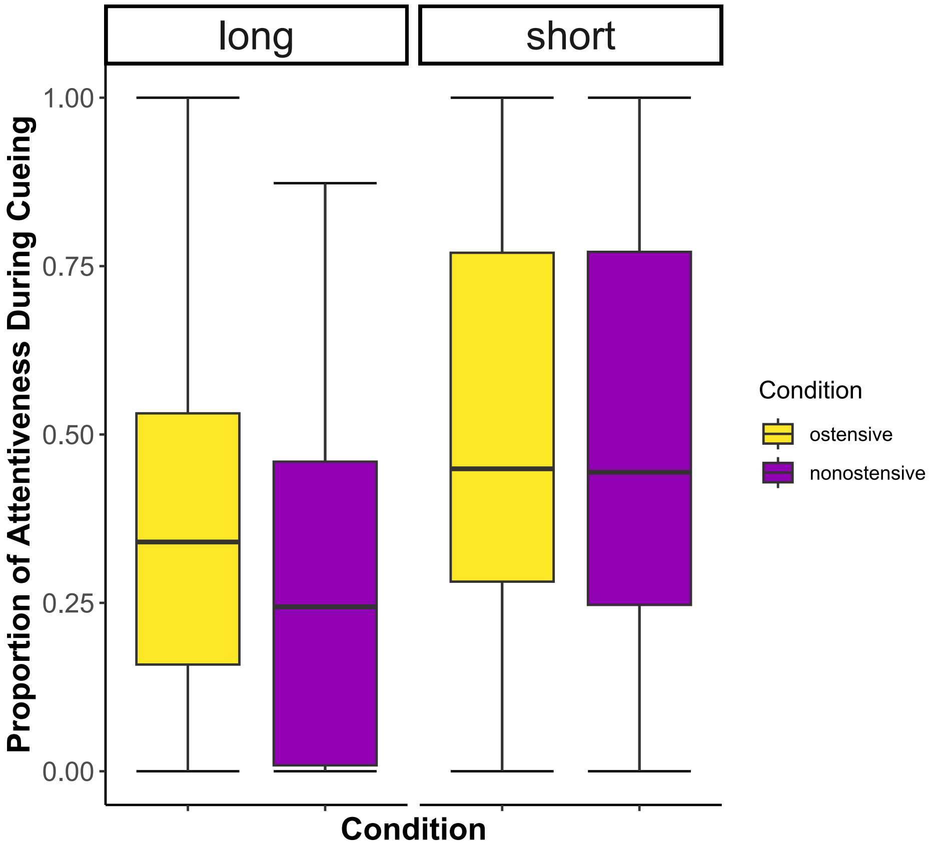

Figure S4: Proportion of attentiveness during the cueing of the object-choice task in the two conditions (ostensive in yellow and non-ostensive in purple) for the pigs that were tested with a short distance and short barrier and those that were tested with a long distance and long barrier.

Succes Object-Choice Task with Directional Gaze and Body Orientation Cues

The success of the two groups in the object-choice task can be seen in Figure S5. Despite subtle differences in success, the non-significant results obtained for the main analysis show that the differences between pigs that experienced a long distance and barrier and those that were tested with a shorter one did not distort the overall conclusion.

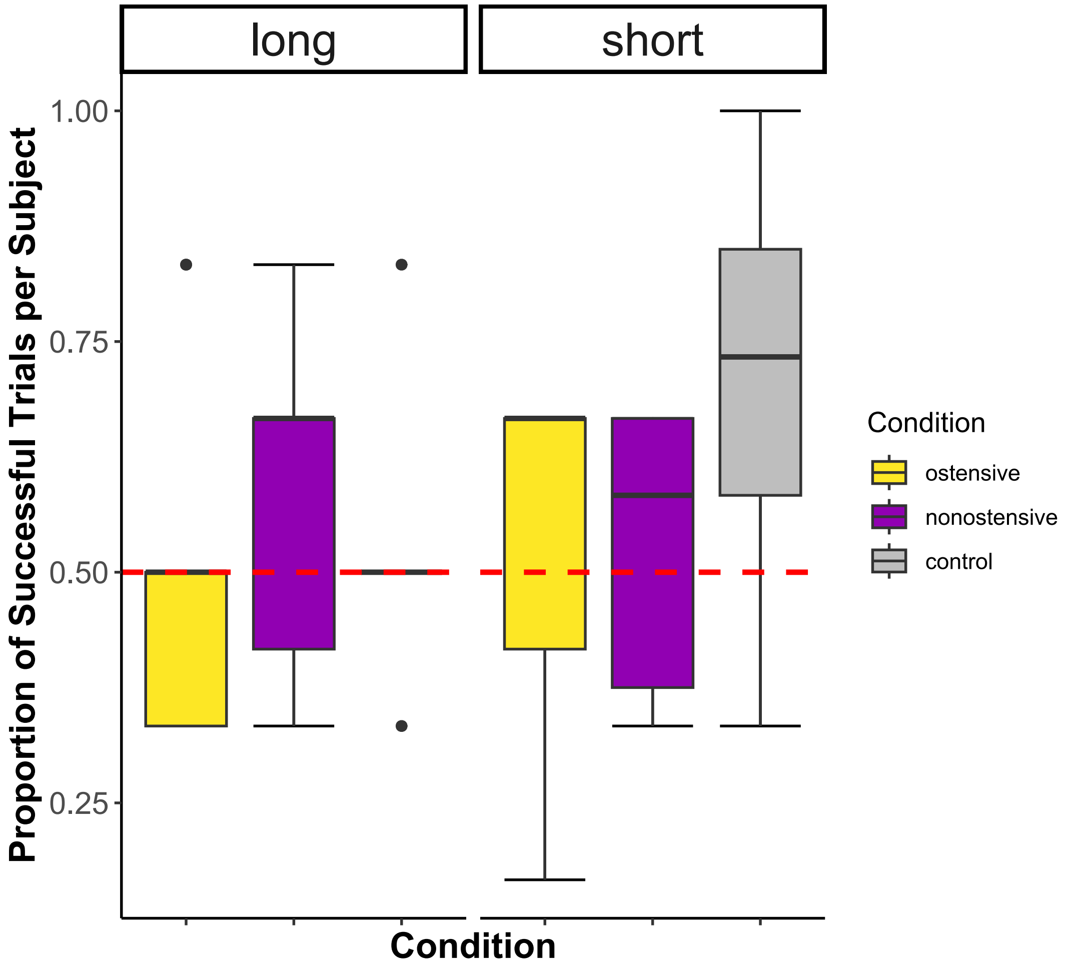

Figure S5: Proportion of successful trials per subject in the object-choice task in the three conditions (ostensive in yellow, non-ostensive in purple, control in gray) for the pigs that were tested with a short distance and short barrier and those that were tested with a long distance and long barrier.

Attentiveness A-not-B Task

The two groups' attentiveness was very similar in the A-not-B task (see Figure S6). Apart from the fact that attentiveness did not significantly differ between conditions in the A-not-B task for the full sample, commercial pigs tested with a shorter barrier show the opposite pattern relative to our predictions. Hence, the difference in distance cannot have biased the overall results towards a more prediction-affirming outcome.

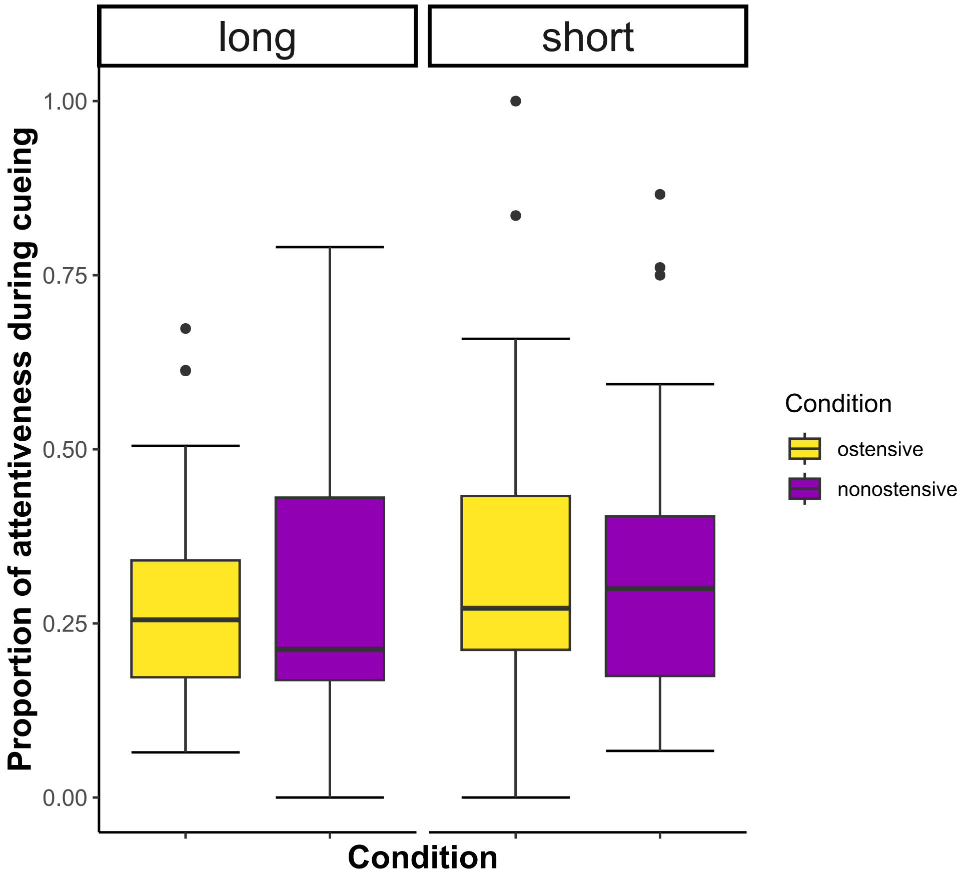

Figure S6: Proportion of attentiveness during cueing in the A-not-B task in the two conditions (ostensive in yellow and non-ostensive in purple) for the pigs that were tested with a short distance and short barrier and those that were tested with a long distance and long barrier.

Success A-not-B Task

Pigs' success in the A-not-B task is visualized in Figure S7. Again, the pigs for which the distance deviated from the proportion used for the other groups exhibit a behavioral pattern that neither conforms with the overall pattern of results for the commercial pigs, nor with our predictions. That is, pigs that were tested with the shorter distance showed a less pronounced A-not-B error in the crucial ostensive B1 trial than those tested with the longer distance (correct proportion).

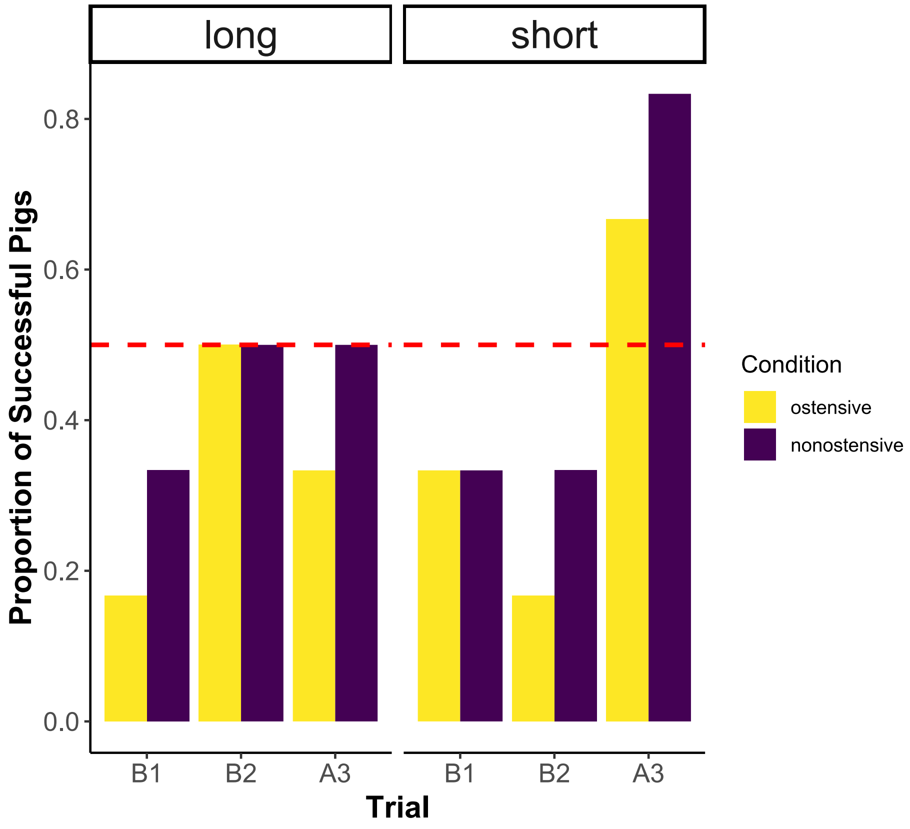

Figure S7: Proportion of successful pigs in the A-not-B task in the two conditions (ostensive in yellow and non-ostensive in purple) for the pigs that were tested with a short distance and short barrier and those that were tested with a long distance and long barrier. The dashed blue line indicates chance level (0.5). Only trials B1, B2 and A3 are depicted as trials A1 and A2 were per definition successful and are, therefore, not relevant to the present comparison.

Conclusions

Given the conclusions we can draw from visual inspection of the differences between the two sub-samples within the commercial pig group, we considered the impact of these divergences on our results and the risk of it leading to false positive results negligible, which is why the commercial pigs were treated as one uniform group in the main analyses without taking unequal barrier and distance lengths into account.

Supplementary Information 3: Model Output Tables and Statistical Details

Attentiveness Object-Choice Task

Table S2: Full model output for the fixed effects of the glmmTMB analyzing the relative attentiveness in the object-choice task (estimates, together with standard errors, significance test statistics, as well as the estimates' confidence limits). P-values are only given for relevant terms.

Term	Estimate	SE	z	X^2	df	P[3]	Lower CI	Upper CI
Intercept	-0.597	0.174	-3.438				-0.937	-0.256
Condition.ostensive[1]	0.152	0.150	1.011	605.22	1	**< 0.001**	-0.143	0.446
Group.companion	1.804	0.301	5.993	54.31	3	0.085	1.214	2.395
Group.lab	1.904	0.230	8.284				1.453	2.354
z.Condition_order[2]	0.003	0.070	0.048				-0.134	0.141
z.Trial_within_Cond[2]	0.079	0.038	2.079				0.004	0.153
Sex.male	-0.243	0.187	-1.300				-0.610	0.124
Condition.ostensive: Group.companion	-0.096	0.268	-0.356	0.22	2	0.893	-0.621	0.430
Condition.ostensive: Group.lab	0.024	0.191	0.125				-0.351	0.398

[1]Condition was dummy coded for the inclusion as a random slope with "nonostensive" being the reference category.

[2]The variables Condition order and Trial within Condition were z-transformed to a mean of 0 and a standard deviation of 1, with the untransformed mean and standard deviation being 2 and 0.817 or 3.5 and 1.709, respectively.

[3]p-value obtained using the drop1 function.

Table S3: Results of pairwise comparisons of pigs' attentiveness between groups in the object-choice task. Significant p-values are printed in bold face.

Pig groups	Estimate	SE	z	p-value
Commercial - Companion	-1.749	0.264	-6.623	**< 0.001**
Commercial - Lab	-1.916	0.203	-9.415	**< 0.001**
Companion - Lab	-0.167	0.226	-0.739	0.740

Attentiveness A-not-B Task

Table S4: Full model output for the fixed effects of the glmmTMB analyzing the relative attentiveness in the A-not-B task (estimates, together with standard errors, significance test statistics, as well as the estimates' confidence limits). P-values are only given for the relevant terms.

Term	Estimate	SE	z	X^2	df	P[3]	Lower CI	Upper CI
Intercept	-0.959	0.204	-4.69				-1.360	-0.559
Condition.ostensive[1]	-0.052	0.145	-0.36	0.572	1	0.449	-0.336	0.233
Group.companion	1.571	0.285	5.15	56.519	2	**< 0.001**	1.013	2.131
Group.lab	2.138	0.229	9.35				1.690	2.587
Trial.A2	0.206	0.116	1.77				-0.022	0.433
Trial.B1	0.281	0.115	2.45				0.056	0.505
Trial.B2	0.290	0.116	2.50				0.062	0.517
Trial.A3	0.317	0.121	2.62				0.080	0.555
z.Session[2]	0.063	0.040	1.58				-0.015	0.141
Sex.male	-0.096	0.180	-0.54				-0.448	0.256
A.side.pigs.view.right	-0.097	0.156	-0.62				-0.402	0.209
Setup.screens	0.118	0.078	1.51				-0.035	0.183
z.WarmUpNumber[2]	0.072	0.057	1.26				-0.039	0.272
Condition.ostensive: Group.companion	0.112	0.235	0.48	0.552	2	0.759	-0.347	0.572
Condition.ostensive: Group.lab	-0.050	0.181	-0.27				-0.405	0.306

[1]Condition was dummy coded for the inclusion as a random slope with "nonostensive" being the reference category.

[2]The variables Session and WarmUpNumber were z-transformed to a mean of 0 and a standard deviation of 1, with the untransformed mean and standard deviation being 1.49 and 0.500 or 1.95 and 3.057, respectively.

[3]p-value obtained using the drop1 function.

Table S5: Results of pairwise comparisons of pigs' attentiveness between groups in the A-not-B task. Significant p-values are printed in bold face.

Pig groups	Estimate	SE	z	p-value
Commercial - Companion	-1.628	0.266	-6.111	**< 0.001**
Commercial - Lab	-2.113	0.210	-10.067	**< 0.001**
Companion - Lab	-0.485	0.218	-2.226	0.067

Attentiveness Detour Task

Table S6: Full model output for the fixed effects of the glmmTMB analyzing the relative attentiveness in the detour task (estimates, together with standard errors, significance test statistics, as well as the estimates' confidence limits). P-values are only given for the relevant terms.

**Term**	**Estimate**	**SE**	**z**	**X²**	**df**	**P[3]**	**Lower CI**	**Upper CI**
Intercept	-0.518	0.222	-2.34				-0.952	-0.084
Condition.nonostensive[1]	-0.461	0.239	-1.93	5.165	1	**0.02**	-0.931	0.007
Group.companion	1.565	0.348	4.50	61.315	2	**< 0.001**	0.884	2.25
Group.lab	2.219	0.268	8.26				1.692	2.745
z.Session[2]	0.058	0.067	0.86				-0.07	0.190
z.Trial.Number	-0.004	0.098	-0.04				-0.196	0.187
Sex.male	-0.319	0.194	-1.64				-0.700	0.062
Setup.mirrorJ	-0.095	0.137	-0.70				-0.364	0.173
Condition.nonostensive: Group.companion	0.100	0.410	0.24	0.650	2	0.772	-0.705	0.904
Condition.nonostensive: Group.lab	0.238	0.299	0.80				-0.349	0.825

[1]Condition was dummy coded for the inclusion as a random slope with "ostensive" being the reference category.

[2]The variables Session and Trial.Number were z-transformed to a mean of 0 and a standard deviation of 1, with the untransformed mean and standard deviation being 1.50 and 0.501 or 4.99 and 0.816, respectively.

[3]p-value obtained using the drop1 function.

Table S7: Results of pairwise comparisons of pigs' attentiveness between groups in the detour task. Significant p-values are printed in bold face.

**Pig groups**	**Estimate**	**SE**	**z**	**p-value**
Commercial - Companion	-1.615	0.282	-5.720	**< 0.001**
Commercial - Lab	-2.338	0.225	-10.385	**< 0.001**
Companion - Lab	-0.723	0.234	-3.084	0.0056

Success Object-Choice Task

Table S8: Full model output for the fixed effects of the glmm analyzing success in the object-choice task (estimates, together with standard errors, significance test statistics, as well as the estimates' confidence limits). No p-values for interactions or single terms were calculated as the full-null model comparison did not yield a significant difference.

Term	Estimate	SE	z	Lower CI	Upper CI
Intercept	0.196	0.311	0.630	-0.414	0.807
Condition.nonostensive	-0.177	0.418	-0.424	-0.996	0.641
Condition.ostensive[1]	-0.180	0.414	-0.434	-0.992	0.632
Group.companion	-0.429	0.485	-0.886	-1.380	0.521
Group.lab	-0.410	0.308	-1.332	-1.014	0.193
z.Condition_order[2]	0.026	0.131	0.202	-0.230	0.2833
z.Trial_within_Cond[2]	-0.073	0.067	-1.094	-0.205	0.058
Side.right	0.180	0.134	1.347	-0.082	0.442
Sex.male	0.003	0.155	0.016	-0.302	0.307
Condition.nonostensive:Group.companion	0.375	0.602	0.622	-0.806	1.555
Condition.ostensive: Group.companion	0.912	0.604	1.509	-0.273	2.096
Condition.nonostensive:Group.companion	0.260	0.400	0.651	-0.523	1.044
Condition.ostensive: Group.lab	0.364	0.394	0.923	-0.408	1.135

[1]Condition was dummy coded for the inclusion as a random slope with "control" being the reference category.

[2]The variables Condition order and Trial within Condition were z-transformed to a mean of 0 and a standard deviation of 1, with the untransformed mean and standard deviation being 2 and 0.817 or 3.5 and 1.709, respectively.

Success A-not-B Task

Table S9: Full model output for the fixed effects of the glmm analyzing success in the A-not-B task (estimates, together with standard errors, significance test statistics, as well as the estimates' confidence limits). P-values are only given for the relevant terms.

**Term**	**Estimate**	**SE**	**z**	**X^2**	**df**	**P[3]**	**Lower CI**	**Upper CI**
Intercept	-1.722	0.597	-2.88				-2.892	-0.551
Condition.nonostensive[1]	0.875	0.649	1.35	0.153	1	0.696	-0.396	2.147
Group.companion	2.161	0.823	2.63	10.675	2	**0.005**	0.548	3.774
Group.lab	0.241	0.593	0.41				-0.922	1.404
Trial.B2	0.927	0.458	2.02	28.269	2	**< 0.001**	0.029	1.826
Trial.A3	1.624	0.465	3.50				0.713	2.535
z.Session[2]	0.239	0.131	1.83				-0.017	0.496
A.side.pigs.view.right	0.102	0.259	0.39				-0.406	0.609
Setup.screens	0.195	0.261	0.75				-0.315	0.609
Sex.male	0.097	0.296	0.33				-0.483	0.706
z.WarumUpNumber[2]	-0.378	0.179	-2.11				-0.729	-0.027
z.Attentiveness_relative[2]	0.043	0.202	0.21				-0.352	0.439
Condition.nonostensive: Group.companion	-1.512	0.900	-1.68	2.943	2	0.230	-3.276	0.252
Condition.nonostensive: Group.lab	-0.419	0.607	-0.69				-1.609	0.771
Condition.nonostensive: Trial.B2	-0.834	0.627	-1.33	2.326	2	0.313	-2.064	0.396
Condition.nonostensive: Trial.A3	-0.049	0.640	-0.08				-1.302	1.205

[1]Condition was dummy coded for the inclusion as a random slope with "nonostensive" being the reference category.

[2]The variables Session, WarmUpNumber and Attentiveness_relative were z-transformed to a mean of 0 and a standard deviation of 1, with the untransformed mean and standard deviation being 1.49 and 0.50, 1.95 and 3.06, or 0.70 and 0.30, respectively.

[3]p-value obtained using the drop1 function.

Table S10: 95%-confidence intervals of pigs' estimated success rate for each combination of condition, pig group and trial in the A-not-B task. Trials in which pigs performed significantly above or below chance level (CIs do not overlap with 0.5) are printed in bold face.

**Pig Group**	**Condition**	**Trial**	**Estimated Probability of Success**	**Lower 95%-CI**	**Upper 95%-CI**
Commercial	Ostensive	B1	0.23	0.07	0.54
Commercial	Ostensive	B2	0.32	0.12	0.62
Commercial	Ostensive	A3	0.48	0.22	0.76
Commercial	Non-ostensive	B1	0.33	0.12	0.63
Commercial	Non-ostensive	B2	0.41	0.17	0.70
Commercial	Non-ostensive	A3	0.67	0.36	0.87
Companion	Ostensive	B1	0.72	0.34	0.93
Companion	Ostensive	B2	0.59	0.19	0.90
Companion	Ostensive	A3	> 0.99	0.00	1.00
Companion	Non-ostensive	B1	0.54	0.22	0.83
Companion	Non-ostensive	B2	0.41	0.14	0.75

Companion	Non-ostensive	A3	0.67	**0.51**	**0.99**
Lab Pigs	Ostensive	B1	0.18	**0.08**	**0.35**
Lab Pigs	Ostensive	B2	0.48	0.30	0.65
Lab Pigs	Ostensive	A3	0.58	0.40	0.74
Lab Pigs	Non-ostensive	B1	0.30	**0.17**	**0.49**
Lab Pigs	Non-ostensive	B2	0.34	0.19	0.53
Lab Pigs	Non-ostensive	A3	0.68	0.50	0.82

Latency to Reach the Target in the Detour Task Between Conditions

Table S11: Full model output for the fixed effects of the Cox mixed effects model analyzing the latency to reach the target in the detour task between conditions. P-values are only given for relevant terms.

**Term**	**coef**	**exp(coef)**	**se(coef)**	**z**	**X^2**	**df**	**P[3]**
Group.companion	1.824	6.199	1.66	1.10	33.218	2	**< 0.001**
Group.lab	2.607	13.558	1.247	2.09			
Condition.nonostensive[1]	0.040	1.040	1.696	0.02	0.006	1	0.938
z.TrialNumber[2]	0.520	1.682	1.020	0.51	8.78	1	**0.003**
z.SessionNumber[2]	0.523	1.686	0.184	2.84			
Setup.mirrorJ	-0.473	0.623	0.313	-1.51			
z.Latency_nodemo_average[2]	-1.065	0.345	0.150	-7.11			
z.Attentiveness_relative[2]	-0.320	0.726	0.154	-2.08			
Sex.male	-0.344	0.709	0.202	-1.70			
Group.companion: Condition.nonostensive	-1.196	0.302	2.417	-0.49	0.398	2	0.820
Group.lab: Condition.nonostensive	-0.211	0.810	1.769	-0.12			
Group.companion: z.TrialNumber	-0.975	0.377	1.550	-0.63	0.257	2	0.880
Group.lab: z.TrialNumber	-0.051	0.950	1.057	-0.05			
Condition.nonostensive: z.TrialNumber	-0.528	0.590	1.566	-0.34	0.625	1	0.429
Group.companion: Condition.nonostensive: z.TrialNumber	2.000	7.390	2.257	0.89	0.791	2	0.673
Group.lab: Condition.nonostensive: z.TrialNumber	0.801	2.228	1.615	0.50			

[1]Condition was dummy coded for inclusion as a random slope with “ostensive” being the reference category.

[2]The variables TrialNumber, SessionNumber, Latency_nodemo_average and Attentiveness_relative were z-transformed to a mean of 0 and a standard deviation of 1, with the untransformed mean and standard deviation being 4.99 and 0.82 (TrialNumber), 1.50 and 0.50 (SessionNumber), 47.44 and 14.91 (Latency_nodemo_average), and 0.68 and 0.29 (Attentiveness_relative).

[3]p-value obtained via comparison of reduced models.

The finding that pigs' latency did not differ between the ostensive and non-ostensive condition (see Table S15) could either mean that both types of demonstrations effectively reduced their latency relative to the no-demonstration trials or that neither of the demonstrations were effective. To discriminate between these two potential explanations, we analyzed whether pigs' latency differed between the no-demonstration trials and the demonstration trials (both ostensive and non-ostensive demonstrations). We predicted that, if demonstrations were effective, the pigs should show a significantly shorter latency in the demonstration trials than in the no-demonstration trials.

We fitted a Cox Mixed Effects Model investigating the effect of the interaction between trial type (no-demonstration or demonstration), session number and pig group on the latency to reach the target, while controlling for trial number (within session, 1–6), set-up (J or mirror-image J) and the pig's sex. Collinearity between fixed effects was not an issue (highest VIF = 1.784). The model was based on 598 observations across 50 subjects.

The full-null model comparison revealed a significant effect of the fixed effects trial type (demo or no-demo), session number and group, or any interaction between the three (n = 598 trials across 50 pigs, χ^2 = 213.93, df = 2, p < 0.001; see Figure S8, for full model output see Table S12). The three-way interaction itself did not have a significant effect (χ^2 = 0.065, df = 2, p = 0.97). Among the two-way interactions, only that between trial type and pig group was significant (χ^2 = 7.111, df = 2, p = 0.03), but not those between trial type and session number (χ^2 < 0.01, df = 1, p = 0.989) and between pig group and session number (χ^2 = 1.295, df = 2, p = 0.52). The pairwise comparisons (Table S13) indicate that both ostensive and non-ostensive demonstrations effectively reduced at least lab pigs' latencies relative to the no-demonstration trials.

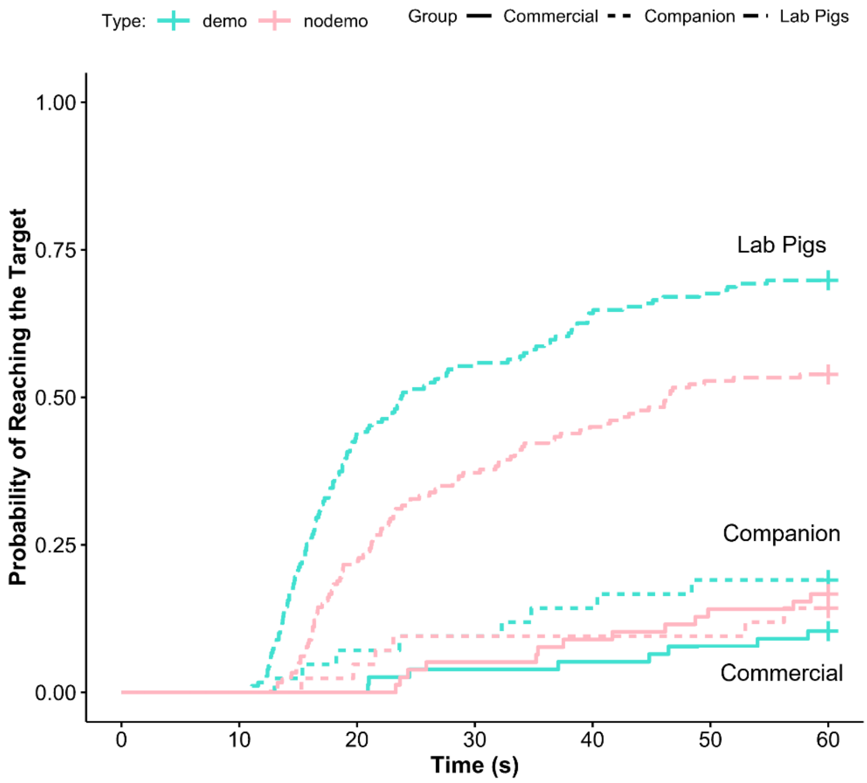

Figure S8: Probability of reaching the target across time (max. 60 s, i.e., the length of a trial) in no-demonstration (light pink) and demonstration (turquoise) trials of the detour task for the three pig groups (Commercial = solid line, Companion = short-dashed line, Lab Pigs = long-dashed line).

Table S12: Full model output for the fixed effects of the Cox mixed effects model analyzing the latency to reach the target in the detour task between the demonstration and no-demonstration trials. P-values are only given for relevant terms.

**Term**	**coef**	**exp(coef)**	**se(coef)**	**z**	**X^2**	**df**	**P[3]**
TrialType.nodemo[1]	0.458	1.581	0.524	0.87			
z.SessionNumber[2]	0.566	1.760	0.409	1.38			
Group.companion	0.386	1.470	0.677	0.57			
Group.lab	2.710	15.160	0.427	6.37			
z.Trial[2]	0.101	1.106	0.063	1.61			
Setup.mirrorJ	-0.008	0.992	0.128	-0.06			
Sex.male	0.052	1.056	0.525	0.39			
TrialType.nodemo: z.SessionNumber	0.054	1.056	0.524	010	< 0.001	1	0.989
TrialType.nodemo: Group.companion	-0.664	0.515	0.928	-0.72	7.111	2	**0.029**
TrialType.nodemo: Group.lab	-1.184	0.306	0.547	-2.16			
z.SessionNumber: Group.companion	0.494	1.639	0.674	0.73	1.295	2	0.523
z.SessionNumber: Group.lab	0.347	1.410	0.421	0.82			
TrialType.nodemo: z.SessionNumber: Group.companion	-0.232	0.793	0.929	-0.25	0.065	2	0.968
TrialType.nodemo: z.SessionNumber: Group.lab	-0.049	0.952	0.548	-0.09			

[1]TrialType was dummy coded for inclusion as a random slope with "demo" being the reference category.

[2]The variables TrialNumber and SessionNumber were z-transformed to a mean of 0 and a standard deviation of 1, with the untransformed mean and standard deviation being 1.997 and 0.816 or 1.501 and 0.500, respectively.

[3]p-value obtained via comparison of reduced models.

Table S13: Results of pairwise comparisons of pigs' latencies for the relevant combinations of pig group and trial type. Significant p-values are printed in bold face.

**Pig group and trial type 1**	**Pig group and trial type 2**	**Estimate**	**SE**	**z**	**p-value**
Commercial - demo	Commercial - nodemo	-0.458	0.524	-0.874	0.960
Companion - demo	Companion - nodemo	0.206	0.765	0.269	0.999
Lab Pigs - demo	Lab Pigs - nodemo	0.725	0.156	4.635	**< 0.0001**
Commercial - nodemo	Companion - nodemo	0.278	0.644	0.432	0.990
Commercial - nodemo	Lab Pigs - nodemo	-1.535	0.358	-4.292	**0.0003**
Companion - nodemo	Lab Pigs – nodemo	-1.814	0.561	-3.230	**0.007**

Supplementary Information 4: Follow-up Analyses of Attentiveness Controlling for Differences in Demonstration Duration

Based on the video coding, we suspected that the length of the cueing/demonstration might have been unequal between conditions. We therefore compared the length of the cueing/demonstration between conditions for each task in a full-null model comparison of Linear Mixed Effects Models. The full models contained the fixed effect condition (ostensive or non-ostensive) and the random effect subject with the random slope of condition (dummy coded), while the null models lacked the fixed effect of condition. The number of included trials and subjects was identical to the respective analyses of attentiveness for each task. Overdispersion was not an issue for any of the models (object-choice task: dispersion ratio = 0.20; A-not-B task: dispersion ratio = 0.35; detour task: dispersion ratio = 0.28).

The cueing duration in the non-ostensive condition was significantly longer than in the ostensive condition for both the object-choice (n = 636 trials across 54 pigs, $\text{mean}_{\text{ostensive}} = 4.18 \pm 0.61$, $\text{mean}_{\text{non-ostensive}} = 4.64 \pm 0.64$, $\chi^2 = 59.06$, $df = 1$, $p < 0.001$) and the A-not-B task (n = 498 trials across 50 pigs, $\text{mean}_{\text{ostensive}} = 8.39 \pm 0.76$, $\text{mean}_{\text{non-ostensive}} = 10.56 \pm 0.83$, $\chi^2 = 128.74$, $df = 1$, $p < 0.001$). Also the non-ostensive detour demonstration was significantly longer than the ostensive one (n = 298 trials across 50 pigs, $\text{mean}_{\text{ostensive}} = 12.35 \pm 1.11$, $\text{mean}_{\text{non-ostensive}} = 13.81 \pm 1.06$, $\chi^2 = 61.73$, $df = 1$, p < 0.001).

To verify that the significant effect of condition on pigs' attentiveness in the detour task and the trend that emerged for the object-choice task cannot solely be attributed to these differences in demonstration/cueing length, we conducted post-hoc analyses in which the demonstration/cueing duration was taken into account. For this purpose, we fitted one additional beta regression model investigating pigs' attentiveness for each task. These models and the procedures were identical to those described in the main manuscript with the exception that the z-transformed duration of the cueing/demonstration was included both as a control predictor and as a random slope within the random intercept of subject. We found that, while the significant effect of condition on pigs' attentiveness in the detour task persisted (n = 298 trials across 50 pigs, $\chi^2 = 9.63$, $df = 1$, $p = 0.002$), a trend was no longer observable for the object-choice task (n = 636 trials across 54 pigs, $\chi^2 = 0.12$, $df = 1$, $p = 0.73$).

Object-Choice Task with Directional Gaze and Body Orientation Cues

In the main analysis investigating pigs' attentiveness in the object-choice task, we observed a tendency for pigs to be more attentive to ostensive than non-ostensive cueing, however, this difference did not reach statistical significance. When taking the cueing duration into account, the full-null model comparison testing the main effects condition and pig group as well as the interaction between the two remained significant ($\chi^2 = 52.87$, *df* = 5, $p < 0.001$). Using reduced models, this difference could be attributed to the significant influence of the main effect group ($\chi^2 = 52.43$, *df* = 2, $p < 0.001$), whereas condition no longer tended to significantly modulate attentiveness ($\chi^2 = 0.112$, *df* = 1, $p = 0.73$). For full model output see Table S14. The new model was not overdispersed (dispersion ratio = 0.219) and the fixed effects were not substantially collinear (highest vif = 1.635).

Table S14: Full model output for the fixed effects of the glmmTMB analyzing the relative attentiveness in the object-choice task, taking the cueing duration into account. P-values are only given for relevant terms.

**Term**	**Estimate**	**SE**	**z**	**X^2**	**df**	**P[3]**	**Lower CI**	**Upper CI**
Intercept	-0.509	0.177	-2.868				-0.856	-0.161
Condition.ostensive[1]	0.082	0.155	0.530	0.12	1	0.728	-0.222	0.386
Group.companion	1.735	0.309	5.620	52.43	2	**< 0.001**	1.130	2.320
Group.lab	1.937	0.236	8.222				1.475	2.399
z.Condition_order[2]	0.030	0.072	0.414				-0.112	0.172
z.Trial_within_Cond[2]	0.071	0.037	1.895				-0.002	0.144
Sex.male	-0.228	0.191	-1.188				-0.603	0.148
z.Cue.duration[2]	-0.173	0.055	-3.181				-0.280	-0.067
Condition.ostensive: Group.companion	-0.191	0.278	-0.688	0.55	2	0.760	-0.737	0.354
Condition.ostensive: Group.lab	-0.042	0.199	-0.208				-0.432	0.349

[1]Condition was dummy coded for the inclusion as a random slope with "nonostensive" being the reference category.

[2]The variables Condition order, Trial within Condition and Cueing Duration were z-transformed to a mean of 0 and a standard deviation of 1, with the untransformed mean and standard deviation being 2 and 0.817, 3.5 and 1.709 or 4.404 and 0.666, respectively.

[3]p-value obtained using the drop1 function.

A-not-B Task

In the main analysis investigating pigs’ attentiveness in the A-not-B task, we did not observe a significant effect of condition (ostension) on pigs’ attentiveness. In the post-hoc analysis, the full model, which included the main effects condition and group as well as the interaction between the two, differed significantly from the null model lacking these terms ($\chi^2 = 56.86$, $df = 5$, $p < 0.001$). The interaction between condition and group did not significantly affect pigs’ attentiveness ($\chi^2 = 0.58$, $df = 2$, $p = 0.746$) and neither did condition ($\chi^2 = 0.16$, $df = 1$, $p = 0.692$). In contrast, we detected significant group differences ($\chi^2 = 55.82$, $df = 2$, $p < 0.001$), which is in line with the results of the main analysis. The full model output can be seen in Table S15. The model was not overdispersed (dispersion ratio = 0.141) and collinearity between the fixed effects was acceptable (highest VIF = 3.677).

Table S15: Full model output for the fixed effects of the glmmTMB analyzing the relative attentiveness in the A-not-B task, taking demonstration duration into account. P-values are only given for the relevant terms.

<table>
<tr><th>Term</th><th>Estimate</th><th>SE</th><th>z</th><th>X^2</th><th>df</th><th>P[3]</th><th>Lower CI</th><th>Upper CI</th></tr>
<tr><td>Intercept</td><td>-1.033</td><td>0.214</td><td>-4.84</td><td></td><td></td><td></td><td>-1.452</td><td>-0.615</td></tr>
<tr><td>Condition.ostensive[1]</td><td>0.092</td><td>0.211</td><td>0.44</td><td>0.157</td><td>1</td><td>0.692</td><td>-0.322</td><td>0.505</td></tr>
<tr><td>Group.companion</td><td>1.611</td><td>0.292</td><td>5.524</td><td rowspan="2">55.816</td><td rowspan="2">2</td><td rowspan="2">< 0.001</td><td>1.039</td><td>2.182</td></tr>
<tr><td>Group.lab</td><td>2.149</td><td>0.235</td><td>9.14</td><td>1.688</td><td>2.610</td></tr>
<tr><td>Trial.A2</td><td>0.224</td><td>0.116</td><td>1.93</td><td></td><td></td><td></td><td>-0.003</td><td>0.451</td></tr>
<tr><td>Trial.B1</td><td>0.245</td><td>0.120</td><td>2.05</td><td></td><td></td><td></td><td>0.010</td><td>0.479</td></tr>
<tr><td>Trial.B2</td><td>0.267</td><td>0.119</td><td>2.24</td><td></td><td></td><td></td><td>0.033</td><td>0.501</td></tr>
<tr><td>Trial.A3</td><td>0.317</td><td>0.121</td><td>2.62</td><td></td><td></td><td></td><td>0.087</td><td>0.562</td></tr>
<tr><td>z.Session[2]</td><td>0.069</td><td>0.044</td><td>1.57</td><td></td><td></td><td></td><td>-0.017</td><td>0.154</td></tr>
<tr><td>Sex.male</td><td>-0.078</td><td>0.182</td><td>-0.43</td><td></td><td></td><td></td><td>-0.433</td><td>0.278</td></tr>
<tr><td>A.side.pigs.view.right</td><td>-0.095</td><td>0.158</td><td>-0.60</td><td></td><td></td><td></td><td>-0.404</td><td>0.214</td></tr>
<tr><td>Setup.screens</td><td>0.140</td><td>0.086</td><td>1.62</td><td></td><td></td><td></td><td>-0.029</td><td>0.308</td></tr>
<tr><td>z.WarmUpNumber[2]</td><td>0.045</td><td>0.059</td><td>0.76</td><td></td><td></td><td></td><td>-0.072</td><td>0.161</td></tr>
<tr><td>z.Demonstration_duration[2]</td><td>0.083</td><td>0.094</td><td>0.89</td><td></td><td></td><td></td><td>-0.100</td><td>0.267</td></tr>
<tr><td>Condition.ostensive: Group.companion</td><td>0.093</td><td>0.260</td><td>0.36</td><td rowspan="2">0.587</td><td rowspan="2">2</td><td rowspan="2">0.746</td><td>-0.417</td><td>0.602</td></tr>
<tr><td>Condition.ostensive: Group.lab</td><td>-0.084</td><td>0.197</td><td>-0.43</td><td>-0.469</td><td>0.302</td></tr>
</table>

[1]Condition was dummy coded for the inclusion as a random slope with “nonostensive” being the reference category.

[2]The variables Session, WarmUpNumber and Demonstration Duration were z-transformed to a mean of 0 and a standard deviation of 1, with the untransformed mean and standard deviation being 1.49 and 0.500 , 1.95 and 3.057 or 9.481 and 1.345, respectively.

[3]p-value obtained using the drop1 function.

Detour Task with Human Demonstrations

The main analysis revealed that the pigs were significantly more attentive to ostensive than non-ostensive detour demonstrations. Also in the post-hoc analyses, the full-null model comparison remained significant ($\chi^2 = 70.48$, $df = 5$, $p < 0.001$), which we could attribute to the main effects of condition ($\chi^2 = 9.63$, $df = 5$, $p = 0.002$) and group ($\chi^2 = 65.24$, $df = 2$, $p < 0.001$), but not the interaction between the two ($\chi^2 = 0.54$, $df = 2$, $p = 0.762$). For full model output see Table S16. The model was not overdispersed (dispersion parameter = 0.121) and there was no substantial collinearity between the fixed effects (highest VIF = 1.959).

Table S16: Full model output for the fixed effects of the glmmTMB analyzing the relative attentiveness in the detour task, taking the demonstration duration into account. P-values are only given for the relevant terms.

Term	Estimate	SE	z	X^2	df	P[3]	Lower CI	Upper CI
Intercept	-0.526	0.219	-2.41				-0.955	-0.098
Condition.nonostensive[1]	-0.636	0.254	-2.50	5.165	1	**0.02**	-1.134	-0.138
Group.companion	1.813	0.363	4.99	61.315	2	**< 0.001**	1.100	2.525
Group.lab	2.382	0.277	8.60				1.840	2.925
z.Session[2]	0.049	0.068	0.73				-0.084	0.182
z.Trial.Number	-0.026	0.098	-0.27				-0.218	0.166
Sex.male	-0.306	0.189	-1.62				-0.677	0.065
Setup.mirrorJ	-0.105	0.138	-0.76				-0.376	0.165
z.Demonstration_duration	0.183	0.086	2.14				0.015	0.35
Condition.nonostensive: Group.companion	0.029	0.414	0.07	0.650	2	0.772	-0.783	0.840
Condition.nonostensive: Group.lab	0.206	0.201	0.69				-0.384	0.796

[1]Condition was dummy coded for the inclusion as a random slope with "ostensive" being the reference category.

[2]The variables Session, Trial.Number and Demonstration Duration were z-transformed to a mean of 0 and a standard deviation of 1, with the untransformed mean and standard deviation being 1.50 and 0.501, 4.99 and 0.816, or 13.08 and 1.30, respectively.

[3]p-value obtained using the drop1 function.

Conclusions

Based on the post-hoc analyses reported above, we can say that the tendency for ostension to enhance pigs' attentiveness in the object-choice task disappears when differences in cueing duration between conditions are taken into account, while the significant effect of ostension on pigs' attentiveness in the detour task persists. In neither of the two analyses did ostension influence pigs' attentiveness in the A-not-B task. Therefore, our finding that pigs' attentiveness to human demonstrations can be enhanced by ostension is not merely an artefact caused by differences in demonstration duration and our main conclusion remains valid.

References Supplementary Material

1. Gerencsér, L., Pérez Fraga, P., Lovas, M., Újváry, D. & Andics, A. Comparing interspecific socio-communicative skills of socialized juvenile dogs and miniature pigs. *Anim Cogn* **22**, 917–929 (2019).
2. Pérez Fraga, P., Gerencsér, L., Lovas, M., Újváry, D. & Andics, A. Who turns to the human? Companion pigs' and dogs' behaviour in the unsolvable task paradigm. *Anim Cogn* **24**, 33–40 (2021).
3. Pérez Fraga, P. Interspecific socio-communicative abilities of the family dog and the family pig from a comparative ethological perspective. (Eötvös Loránd University, 2023).
4. Pérez Fraga, P., Morvai, B., Gerencsér, L., Lehoczki, F. & Andics, A. Out-of-reach rewards elicit human-oriented referential communicative behaviours in family dogs but not in family pigs. *Sci Rep* **13**, 811 (2023).
5. Veit, A., Fuxjäger, I., Wondrak, M. & Huber, L. Social influence and attention bias in free-ranging domestic pigs: Effects of demonstrator rank and friendship. *Applied Animal Behaviour Science* **275**, 106285 (2024).
6. Blakey, K. H. *et al.* Non-verbal rationality? 2-year-old children, dogs and pigs show unselective responses to unreliability, but to different degrees. Preprint at https://doi.org/10.31234/osf.io/ajh76 (2024).
7. Veit, A., Weißhaupt, S., Bruat, A., Wondrak, M. & Huber, L. Emulative learning of a two-step task in free-ranging domestic pigs. *Anim Cogn* (2023) doi:10.1007/s10071-022-01740-3.
8. Nestelberger, M. Problem-solving ability in free ranging Kune Kune pigs (*Sus scrofa domesticus*) according to their position in the social network. (2019).
9. Studnitz, M., Jensen, M. B. & Pedersen, L. J. Why do pigs root and in what will they root?: A review on the exploratory behaviour of pigs in relation to environmental enrichment. *Applied Animal Behaviour Science* **107**, 183–197 (2007).
