# Supplementary material for: Human ostension enhances attentiveness but not performance in domestic pigs
Source: Sci Rep. 2025 May 9;15:16161. doi: 10.1038/s41598-025-00511-7 (PMC12064724; doi:10.1038/s41598-025-00511-7)
Supplement: Supplementary file 5 — Supplementary Material 5 [file 41598_2025_511_MOESM5_ESM.docx]

Video titles and legends to „ Human Ostension Enhances Attentiveness but Not Performance in Domestic Pigs”

Supplementary Video 1:

Title: Object-Choice Task

Caption: Example trials of the object-choice task for the three conditions (ostensive, non-ostensive, control) and the three pig groups

Supplementary Video 2:

Title: A-not-B Task

Caption: Example trials of the A-not-B Task showing one A-trial and one B-trial per condition (ostensive and non-ostensive)

Supplementary Video 3:

Title: Detour Task

Caption: Example trials of the detour task, showing a no-demonstration trial as well as one ostensive and one non-ostensive demonstration trial
